# Nanocarbon Reinforced Rubber Nanocomposites: Detailed Insights about Mechanical, Dynamical Mechanical Properties, Payne, and Mullin Effects

**DOI:** 10.3390/nano8110945

**Published:** 2018-11-16

**Authors:** Suneel Kumar Srivastava, Yogendra Kumar Mishra

**Affiliations:** 1 Inorganic Materials and Polymer Nanocomposite Laboratory, Department of Chemistry, Indian Institute of Technology, Kharagpur-72102, India; 2 Functional Nanomaterials, Institute for Materials Science, Kiel University, Kaiserstr, D-24143 Kiel, Germany

**Keywords:** rubber nanocomposites, mechanical properties, dynamical mechanical properties, Payne effect, Mullin effect

## Abstract

The reinforcing ability of the fillers results in significant improvements in properties of polymer matrix at extremely low filler loadings as compared to conventional fillers. In view of this, the present review article describes the different methods used in preparation of different rubber nanocomposites reinforced with nanodimensional individual carbonaceous fillers, such as graphene, expanded graphite, single walled carbon nanotubes, multiwalled carbon nanotubes and graphite oxide, graphene oxide, and hybrid fillers consisting combination of individual fillers. This is followed by review of mechanical properties (tensile strength, elongation at break, Young modulus, and fracture toughness) and dynamic mechanical properties (glass transition temperature, crystallization temperature, melting point) of these rubber nanocomposites. Finally, Payne and Mullin effects have also been reviewed in rubber filled with different carbon based nanofillers.

## 1. Introduction

Recently, one-dimensional (1D) carbon nanotubes (CNTs) and two-dimensional (2D) graphite oxide and graphene constitute proven graphitic materials receiving considerable attention [1,2,3,4,5,6,7,8,9,10]. This is mainly attributed to their outstanding properties, such as larger surface area, excellent thermal, mechanical, electrical, and optical properties. In spite of their identical chemical composition, graphene exhibits added advantage due to the abundant availability of graphite as a naturally occurring precursor and its production cost as compared to CNTs. These graphitic materials find applications in the field of sensors, batteries, solar cells, actuators, supercapacitors, catalysis, organic light-emitting diodes, field emission transistors, and optical/electrochemical devices, etc. A recent study has shown that these graphitic materials can be successfully used as effective nanofillers in polymer even at very small loadings in order to significantly improve properties of polymers. However, agglomeration remains one of the most common problems with CNTs as well as graphene due to the presence of inter-tubular interaction and the restacking of graphene sheets, respectively. In addition, their poor dispersion in many common organic solvents as well as in polymer matrices remains another issue. Such restacking tendency of these graphitic fillers could be overcome through their surface, leading to the better dispersion of filler and polymer through strong filler filler-polymer interaction. Graphite oxide [5,11] and carbon nanofibre [7] are other carbon materials investigated in the development of polymer nanocomposites. Recently, (3D) materials that are obtained by the hybridization of 1D and 2D graphene have also been employed as hybrid fillers due to enhanced dispersion as compared to the dispersion problem faced by the individual fillers in polymers [12,13].

**Scheme 1:**
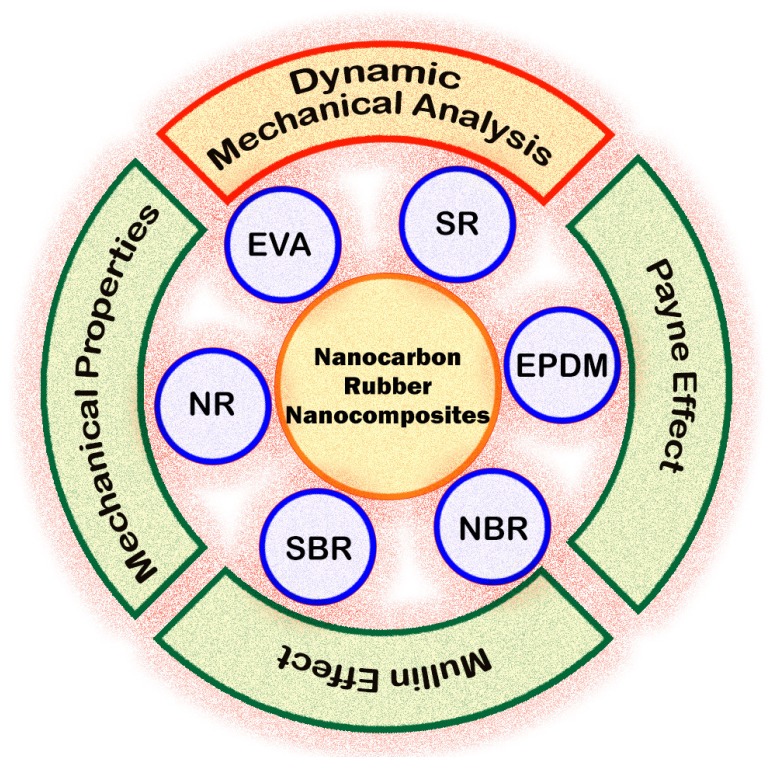
Schematic representation showing different nanocarbon rubber nanocomposites mechanical and dynamical properties, Pyane and Mullin effects.

Elastomers, according to the general IUPAC definition, are polymers that exhibit rubber-like elasticity. They find extensive applications in a variety of commercial as well as domestic products [14,15,16,17,18,19]. The most common examples of these elastomers are natural rubber (NR). In addition, other important rubbers include silicone rubber (SR), styrene butadiene rubber (SBR), acrylonitrile butadiene rubber (NBR), butyl rubber (BR), ethylene vinyl acetate copolymer (EVA), and ethylene propylene diene rubber (EPDM), etc. Among those different rubber types available, NR is largest single type produced from latex, whereas others are synthetic polymers that are manufactured either to replace or to be used together with NR or to make polymers with properties superior of NR. The high deformability (viscoelastic behavior) of these elastomers remained one of most desirable properties for their industrial applications. However, their low elastic modulus and durability makes it mandatory to use carbon black (CB), clay, graphite, and silica, etc. as fillers. The unique properties of the rubber composites filled with carbon nanofillers have attracted industry. In view of this, these carbon containing fillers has been receiving considerable amount of attention as filers in rubber nanocomposites due to their superior properties. When considering all these aspects, the present article reviews mechanical properties, dynamical mechanical properties, Payne, and Mullin effects of NR, SBR, NBR, EPDM, and EVA rubbers filled with graphitic fillers anticipating their superior applications in multifaceted fields [20,21,22,23,24,25,26,27,28,29,30,31,32,33,34,35,36,37,38,39,40,41,42,43,44,45,46,47,48,49,50,51,52,53,54,55,56,57,58,59,60,61,62,63,64,65,66,67,68,69,70,71,72,73,74,75,76,77,78,79,80,81,82,83,84,85,86,87,88,89,90,91,92,93,94,95,96,97,98,99,100,101,102,103,104,105,106,107,108,109,110,111,112,113,114,115,116,117,118,119,120,121,122,123,124,125,126,127,128,129,130,131,132,133,134,135,136,137,138,139,140,141,142,143,144,145,146,147,148,149,150,151,152,153,154,155,156,157,158,159,160,161,162,163,164,165,166,167,168,169,170,171,172,173,174,175,176,177,178,179,180,181,182,183,184,185,186,187,188,189,190,191,192,193,194,195,196,197,198,199,200,201,202,203,204,205,206,207,208,209]. The main objectives in this this review article which could be of interest for readers, are schematically demonstrated by the Scheme 1 as below.

## 2. Preparation and Methodology of Rubber Nanocomposites

Rubber nanocomposites have been invariably prepared by solution blending and melt blending. The solution blending method of preparation is not eco-friendly and cost-effective due to the use of excess amount of organic solvents. On the contrary, melt-mixing of polymer with nanofiller is the most effective way to obtain the nanocomposites for commercial application. The method is totally environmentally friendly and does not require solvent. Any kind of polymers, such as thermoplastic or thermosetting, can be used to prepare nanocomposites by these techniques. It may be noted that properties of polymer nanocomposites are determined by the dispersion of the filler(s) in the polymer matrix and also depend on polymer-filler interactions. In view of this, different methods used in fabrication of polymer nanocomposites by mechanical blending, solution blending, in-situ polymerization, etc. could result in varying degrees of dispersion, as evidenced from scanning electron micrographs (SEM), field emission SEM, transmission electron microscopy (TEM), and high resolution TEM. Table 1, Table 2, Table 3 and Table 4 records the carbon-based fillers, methodology followed and morphology achieved for NR, SBR, NBR, and SR rubber nanocomposites.

## 3. Mechanical Properties of Rubber Nanocomposites of Carbon Based Fillers

The performance of a composite generally depends on the various filler parameters, including geometry, stiffness, and orientation. In addition, the dispersion of CNT, CNF, graphite oxide, and graphene within the rubber matrix account for enhanced properties of rubber. The effect of carbon based nanofiller incorporated rubber nanocomposites are described, as below.

### 3.1. Mechanical Properties of Carbon Filler Incorporated NR and Its Blend Nanocomposites

NR is an important unsaturated elastomer and a polymer of isoprene. It possesses high strength, high tear resistance, low heat build-up, high resilience, and retention of strength at elevated temperature, excellent dynamic properties and general fatigue resistance. It demonstrated excellent chemical and physical properties, including high elasticity and flexibility, corrosion resistance, antivirus permeation, and biodegradability [14]. NR finds extensive applications either alone or in combination with other materials in the transportation (e.g., tyres), industrial (sealants), consumer (sports materials), hygienic, and medical sectors. Despite its extreme flexibility and stretchable properties, it is subject to weathering and has a poor resistance to heat oil and ozone, despite being generally waterproof. Therefore, it is anticipated that carbon nanofillers and their nanohybrids reinforced NR and NR blend nanocomposites could find better applications in tires, adhesives, surgical gloves, and sealing materials, etc. due to their superior properties.

**Figure 1:**
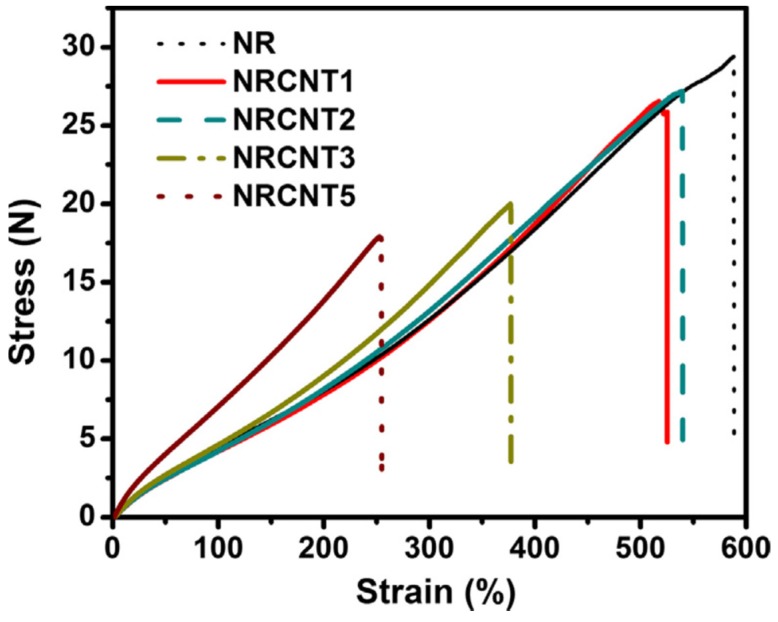
Stress-strain curves for pure NR and NR/multiwall carbon nanotubes (MWCNT) composites. Reproduced with permission from Elsevier [23].

Mechanical properties of different carbon filler based natural rubber nanocomposites has been studied by many workers [20,21,22,23,24,25,26,27,28,29,30,31,32,33,34,35,36,37,38,39,40,41,42,43,44,45,46,47,48,49,50,51,52,53,54,55,56,57,58,59,60,61,62,63,168,169,170,171,172,173,174,175,176,177,178,179,180,181,182,183,184,185,186,187,188,189,203,204,205]. Tarawneh et al. [22] investigated the mechanical properties of thermoplastic natural rubber (TPNR) nanocomposites reinforced by multiwall carbon nanotubes (MWCNTs), which showed that 3 wt % of MWCNTs loading in TPNR resulted in increase of ~39% and 30% in tensile strength and Young’s modulus, respectively. Also, it was noted that elongation at break decreased with increase in the percentage of MWCNTs. The maximum impact strength was recorded at 5 wt % of MWCNTs which was increased by 74% as compared with a pristine TPNR. Ponnamma et al. [23] reported stress–strain curves for pure NR and NR/MWCNT composites, as shown in Figure 1. It is noted that NR exhibits a large increase in stress at higher deformations due to strain-induced crystallization, whereas, the strain at rupture is reduced for its MWCNT composites. The corresponding mechanical data of NR/MWCNT nanocomposites shows increase in Young’s modulus of NR (~0.50 MPa) with MWCNT loadings. It was found to attain highest value of ~1.10 MPa at 5 wt % of maximum MWCNT loading. This is attributed to the anisometry of the filler structures, its nucleating effect, and dispersion of filler and polymer-filler interactions. They also observed maximum improvement in tensile strength at around 1 wt % loading of MWCNT in NR, whereas, elongation at break showed no improvement on MWCNT loading in NR. Tarawneh et al. [24] studied the effect of sonication on the mechanical properties of TPNR nanocomposites reinforced by 1 wt % of MWCNT. The Young’s modulus, tensile strength, elongation at break, and impact strength increased by almost 11%, 21%, 43%, and 50%, respectively, as compared with a pristine due to good dispersion achieved after optimal sonication time of 1 h.

Nakaramontri et al. [26] used ex-situ and in-situ surface functionalized MWCNT as filler in NR and studied their mechanical properties. Table 5 shows the tensile strength, elongation at break, and 100% modulus data of NR gums and MWCNT composites of melt and latex-based samples. Addition of CNTs resulted in an increase of 100% modulus and a decreasing elongation at break due to the reinforcement effect of MWCNTs. Tensile strength decreased on adding either raw MWCNTs or ex-situ functionalized MWCNTs to the NR. In contrast, in-situ functionalization exhibited higher tensile strength when compared to neat rubbers. Elongation at break values of NR composites filled with raw or ex situ-functionalized MWCNTs were found to be lower than unfilled rubbers. Table 5 also revealed the superior tensile tests performance of composites prepared by in situ method. Ali and Ahmad [27] studied mechanical properties of polylactic acid (PLA)/liquid natural rubber (LNR) filled with MWCNT prepared by melt blending method. This result has shown that PLA/LNR nanocomposite filled with 3.5 wt % of MWCNTs exhibited 22 and 20% higher tensile strength and Young’s modulus, respectively. Such improvements could be directed to the good dispersion of MWCNTs inside the PLA/LNR matrix. The investigations also indicated decrease in elongation at break of PLA/LNR with increasing amount of MWCNTs. The impact strength also increased to ~42% in PLA/LNR/MWCNTs (3.5 wt %) due to the better dispersion of MWCNTs in the matrix generating significant toughening effect. Anand et al. [30] observed significantly improved the mechanical properties in NR/SWCNTs nanocomposites prepared through a latex stage mixing method. NR/SWNTs (2.0 phr) in relation to pure NR containing SWCNTs showed tensile strength and tensile modulus higher by 56% and 63%, respectively.

Mechanical properties of NR/MWCNT (2 to 25 phr) nanocomposites prepared by ultrasonically aided extrusion at ultrasonic amplitudes up to 7.5 µm have been studied as a function of ultrasonic amplitude at various filler loadings [31]. It is observed that the tensile strength of NR/CNT vulcanizates decreases or does not change with increasing ultrasonic amplitude, though, elongation at break is slightly affected by ultrasonic treatment. Also, hardness of NR/CNT vulcanizates at loadings up to 15 phr remains more or less unaltered with increasing ultrasonic amplitude. This is ascribed mainly due to the chain scission and/or loss of double bonds during ultrasonic treatment. Kueseng and Jacob [33] also studied mechanical properties of single-walled carbon nanotubes (SWCNT) filled NR nanocomposites that were prepared by solvent method. The corresponding mechanical test results show an increase in the initial modulus for up to 50% in relation to pure NR. The modulus also increased with increasing SWCNT contents. Tarawneh et al. [38] also determined the optimum mechanical properties of different percentages of MWCNTs-OMMT (1 wt % MWCNTs + 3 wt % OMMT, 2 wt % MWCNTs + 2 wt % OMMT, and 3 wt % MWCNTs + 1wt % OMMT) hybrid filled TPNR nanocomposites. These findings showed increase in tensile strength and Young’s modulus in the presence of nanotubes. The corresponding values of ~33% and 36% were obtained in 3 wt % nanotubes filled TPNR when compared with pure TPNR matrix. Such significant improvement in these mechanical properties was assigned to interfacial adhesion between fillers and the matrix due to aspect ratio and fillers orientation in the TPNR matrix. In contrast, elongation at break considerably decreased with increasing the percentage of MWCNTs.

Melt blended ENR/MWCNT and the ENR/MWCNT modified by bis(triethoxysilylpropyl) tetrasulfide (TESPT) composites exhibit improved tensile properties and Young’s modulus than pristine ENR [41]. Azira et al. [42] prepared MWCNT/ENR nanocomposite through latex technology and observed a considerable increase in its mechanical comparison to either neat ENR and its CB filled ENR composite. Natural rubber composites with different contents of 1, 3, 10, and 20 wt % vapor-grown carbon nanofibers (VGCFs) were synthesized using a solvent casting method [43]. Their mechanical property investigations showed NR/3 wt % VGCF composite had the greatest tensile strength. The stress-strain curves of NR/TRG composites clearly indicate that there is a dramatic improvement (282%) in tensile modulus at low loading (3% wt./wt.) of TRG [203].

**Figure 2:**
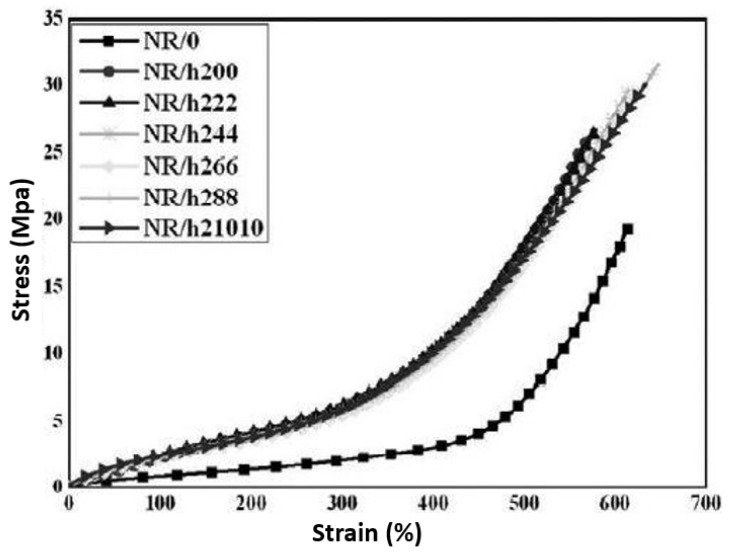
Stress-strain curves of NR/0 and different NR/MWCNT/expanded organically modified montmorillonite (EOMt) nanocomposites. Reproduced with permission from [44], Copyright Elsevier, 2015.

**Figure 3:**
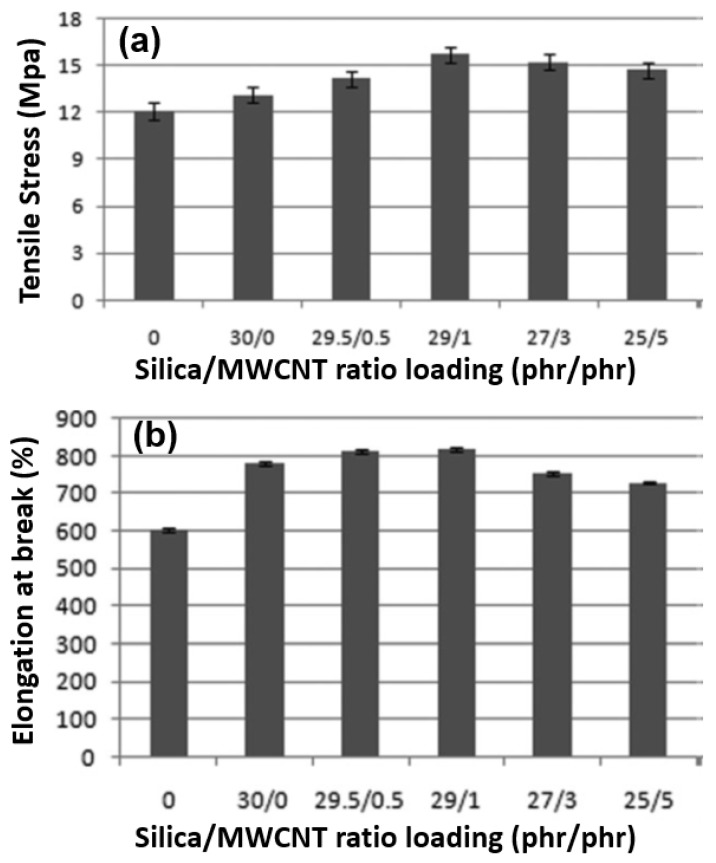
(**a**) Effect of the silica/MWCNT hybrid loading ratio on the tensile strength of the NR/silica/MWCNT hybrid nanocomposites; (**b**) Effect of the silica/NWCNT hybrid loading ratio on the EB of the NR/silica/MWCNT hybrid nanocomposites. Reproduced with permission from [34], Copyright Wiley, 2013.

**Figure 4:**
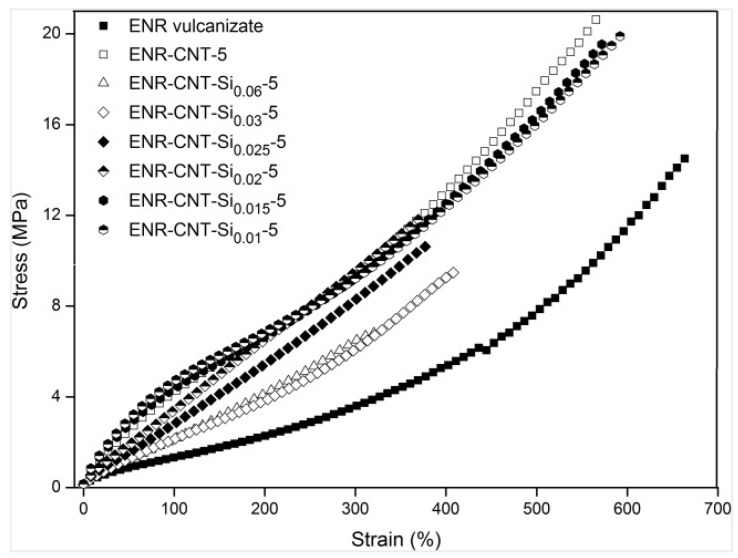
Stress-strain curves of epoxidized natural rubber (ENR)vulcanizate and ENR-CNT composites without and with APTES at concentrations from 0.06 to 0.01 mL/(g of CNTs). Reproduced with permission from [40], Copyright Wiley 2017.

Hybrid nanomaterials, such as MWCNT/Silica, [20] MWCNT/CB, [32,35,54], CNT-clay, [38] CNT-Graphene [39] have also been used in reinforcing of natural rubbers. Fritzsche et al. [20] incorporated MWCNTs in highly silica filled NR by applying melt mixing techniques. To distinguish between properties that are based on silica and MWCNT, the amount of silica has been successively exchanged by the same amount of MWCNTs upto 10 phr of MWCNT. The resulting samples show an increased mechanical stiffness and tensile strength. The tensile strength increases from 14 to 17 MPa when part of the silica is exchanged by 10 phr of MWCNT, though the elongation at break is successively reduced. Stress–strain behavior of NR composites with silica and CNT (dry mixing) showed mechanical reinforcement of the CNT/silica hybrid system at strains below 100% mainly results from the CNT [24]. Xu et al. [32] prepared NR/MWCNTs (1–5 phr) master batch by latex compounding assisted by anionic surfactants containing phenyl ring moieties and co-coagulated process and measured their mechanical properties. It is noted that the tensile strength, stress at 300% strain, and tear strength of NR/CB/MWCNTs and composite with 4 phr MWNTs loading were increased by ~12.3%, 26.0%, and 16.9%, respectively. Such improvement in mechanical properties is ascribed to the homogenous dispersion of MWNTs, interfacial adhesion between MWNTs and NR polymer chains. Alternatively, possibility synergistic reinforcing effect of CB and MWNTs in NR matrix also cannot be ruled out. Dong et al. [35] studied mechanical properties of natural rubber composites reinforced with hybrid fillers consisting of carbon nanotube bundles (CNTB)/CB (0/25, 1/22, 3/16, 5/10 phr) and the findings are displayed in Table 6. It is noted that CNTB/CB (5/10 phr) hybrid filled NR composite showed remarkable increments in the stress at 100% and 300% corresponding to 64% and 57% increase in stress compared to neat natural rubber. The tensile strength and tear strength were also improved, indicating an enhanced fracture resistance with increasing content of CNTBs. They ascribed such reinforcement of the CNTBs due to high aspect ratio and surface area. In another study, stress-strain plots of NR filled with hybrid consisting of MWCNT (2 phr) and expanded (0–20 phr) organically modified montmorillonite (EOMt) were studied, as shown in Figure 2 [44]. They noted significant improvement of the tensile modulus for all nanocomposites due to the strong interaction of MWCNT with the elastomeric matrix. Further, tensile strength attained maximum improvements in NR/MWCNT-2 phr (25.84 MPa) as compared to that of neat NR (22.38 MPa). The addition of EOMt further increases tensile strengths of NR/MWCNT-2 phr/EOMT-16 phr (31.15 MPa), followed by a slight decrease in NR/MWCNT-2phr/20phr-/EOMT nanocomposite (29.12 MPa). Further, the nanocomposites consisting 16 and 20 phr/EOMt exhibit not only higher modulus but also a slightly higher elongation at break (EB). Ismail et al. [34] studied the effects of SiO_2_/MWCNT hybrid filler (total loading fixed at 30 phr) on the mechanical properties of NR nanocomposites and findings are displayed in Figure 3. It is seen that silica/MWCNT hybrid loading (29/1) exhibits highest tensile strength and EB due to the good dispersion and lower agglomeration of both fillers. However, tensile strength and EB decrease at further higher MWCNT loading ratio due to poor filler- rubber interaction in the silica/MWCNT hybrid. This study also indicated steady increase in both M100 and M300 with MWCNT loading ratio increased in the silica/MWCNT hybrid. Such observations indicated that the addition of MWCNTs improved the stiffness of the nanocomposites. Tensile properties of ENR-CNT composites prepared by in situ functionalization with various APTES silane concentrations were investigated [40]. Figure 4 shows stress-strain curves of ENR vulcanizate and ENR-CNT composites without and with APTES at concentrations from 0.06 to 0.01 mL/(g of CNTs). It is clearly seen that tensile and the tensile strength rapidly increased with the addition of CNTs in the ENR matrix. This is due to the chemical interactions of polar functional groups in ENR with the CNT surfaces. In contrast, the tensile strengths of the ENR-CNT composites with APTES were lower than without APTES.

Wipatkrut and Poompradub [177] studied the effect of graphite oxide reduced by l-ascorbic acid in NR on the mechanical properties of resulting composites. The tensile strength and hardness were found to be comparable with those of conductive carbon black filled NR.NR/PVP (5 phr) nanocomposites exhibited enhanced tensile strength (81%) and tear strength (159%) as compared with pristine NR [178]. The enhanced mechanical properties were also achieved in NR/GO [83,84,85] and NR/TRGO (thermally reduced graphite oxide) [182,183]. Yan et al. [184] reported that tensile strength and tensile modulus at 300% strain of NR/HDPE/GO (1.5 phr) as compared to neat blend were increased by ~27% and ~24%, respectively. The effects of GO content on the mechanical properties of NR-g-GMA/GO nanocomposites were investigated [185]. The tensile strength and tear strength of the 3 phr GO loaded in NR-g-GMA with showed maximum improvements when compared with NR/GO nanocomposites. Wu et al. [186] reported that maleic anhydride grafted liquid polybutadiene functionalized graphene oxide (MLPB-GO) filled NR composites that were prepared by co-coagulation process composites are obviously superior to those of NR/GO composites and neat NR. They found that tensile strength, modulus at 300% strain and tear strength of NR composite containing 2.12 phr MLPB-GO compared with neat NR are increased by 40.5%, 109.1%, and 85.0%, respectively. The significant reinforcement of MLPB-GO in NR is ascribed to the good dispersion of GO and the strong interface interaction in the composites. 

Graphene has also been used as reinforcing filler in natural rubber as evident from its mechanical properties [5,33,34,48,49]. Galimberti et al. [36] recorded nominal stress-nominal strain curves on nanocomposites of synthetic poly(1,4-cis-isoprene) (IR) filled with organoclay, CB, MWCNT, and graphene. The appreciably larger elongation at break was observed in IR/MWCNT compared to those in IR/CB nanographene. Recently, investigations related to mechanical properties of the unfilled NR, graphene filled NR, CNT filled NR, and NR/MWCNT/graphene has been made [39]. It is observed that 0.5 phr graphene filled NR remarkably increases the tensile strength of NR as compared to unfilled NR (17.8 MPa). Interestingly, hybridizing MWCNTs with graphene contributed significant reinforcement effect in NR. NR/FGSs nanocomposites prepared by two-roll mill mixing showed enhancement on the mechanical behavior due to strong rubber-to-filler interactions [49]. 

**Figure 5:**
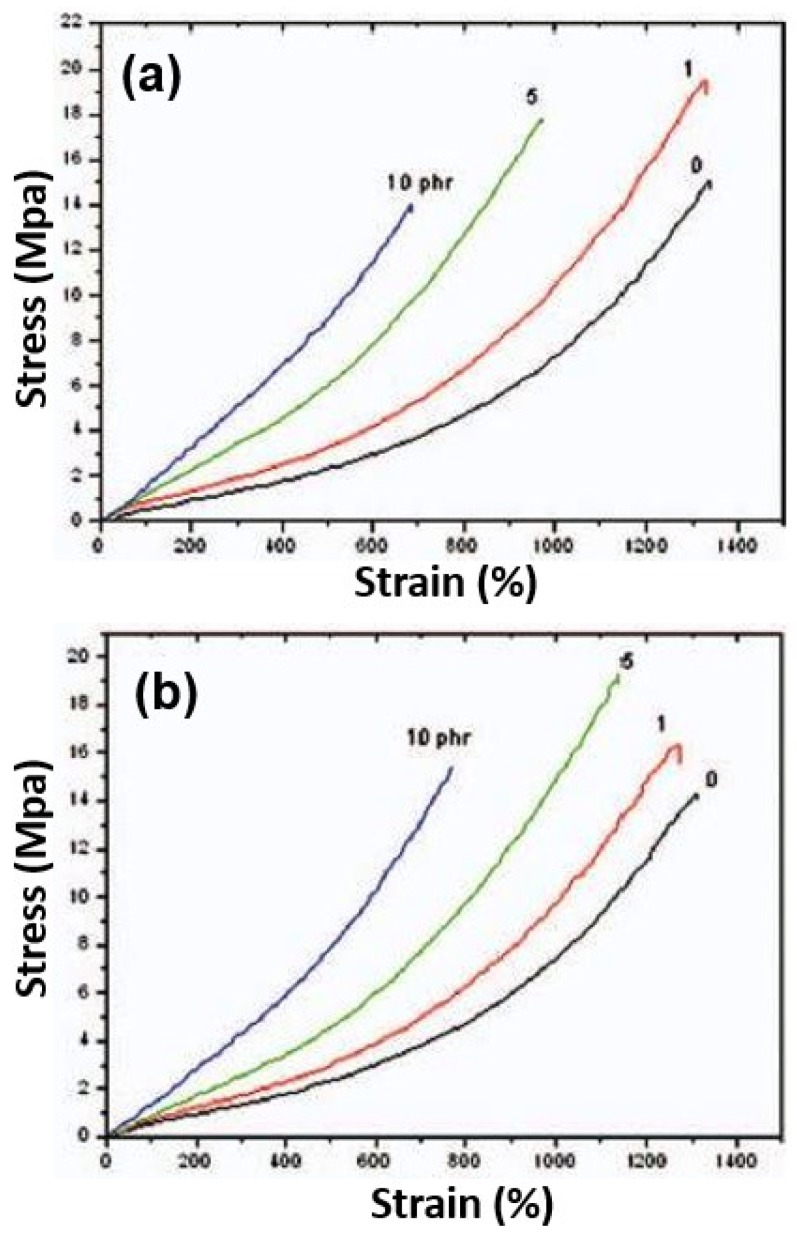
Stress-strain curves of NR composites with (**a**) CNT and (**b**) phenol functionalized CNT. Reproduced with permission from [62], Copyright Wiley, 2012.

Thomas et al. [62] studied the effect of phenol functionalization of carbon nanotubes on the properties of natural rubber nanocomposites on stress-strain behavior, as displayed in Figure 5. It is noted that tensile strength of the composites increases on incorporating of 1 phr of CNT, while higher loading of CNT causes a decrease in tensile strength. Incorporation of CNT causes an increase in Young’s modulus of the composites at all loadings, while the elongation at break decreases at higher filler loadings. When CNT is functionalized with phenol and used as filler in NR, an increase in Young’s modulus is observed at all loadings. Further, it is noted that tensile strength attains a maximum value at a 5 phr loading and beyond which it decreases, while the elongation at break shows a gradual decrease with increase in CNT loading. The tensile strength, modulus at 300% elongation, and tear strength for NR composites containing 0.9 phr of reduced graphene as compared to NR increased by 50.2%, 154.9% and 65.2%, respectively [187]. The incorporation of ZnO nanoparticles doped graphene (5 phr) in NR matrix showed significantly improved mechanical properties over that of NR composite filled with conventional-ZnO [188]. In this context, recently developed carbon based complex hierarchical nanostructures, ZnO tetrapod, 3D interconnected network/hybrids could play a very important role as filler in materials towards development of advanced rubber composites with engineered properties NR and other rubbers [210,211,212,213,214,215,216,217,218,219,220,221,222].

The incorporation of 0.5 phr of graphene in NR was used to prepare corresponding nanocomposites by a modified latex mixing method combined with in situ chemical reduction [189]. The 48% increase in the tensile strength and an 80% increase in the initial tensile modulus are achieved without sacrificing the ultimate strain. But, further increasing the GE loading degrades the tensile strength and the ultimate strain. But, further increasing the GE loading degrades the tensile strength and the ultimate strain. However, more work on fabricating rubber nanocomposites consisting other carbon nano fillers, such as SWCNT, expanded graphite needs to be undertaken in future work.

### 3.2. Mechanical Properties of Carbon Filler Incorporated SBR and Its Blend Nanocomposites

SBR exhibit better processability, heat aging, and abrasion resistance, but it is inferior in terms of elongation, hot tear strength, hysteresis, resilience, and tensile strength. NBR is widely used due to great oil resistance, low gas permeability, and high shear strength. The varying ratio of nitrile within the polymer can change these characteristics. The addition of carbon containing fillers could enhance the processability and mechanical and many other properties of SBR [64,65,66,67,68,69,70,71,72,73,74,75,76,77,78,79,80,81,82,83,84,85,86,87,88,89,90,190,191,192,193,194,206,207].

**Figure 6:**
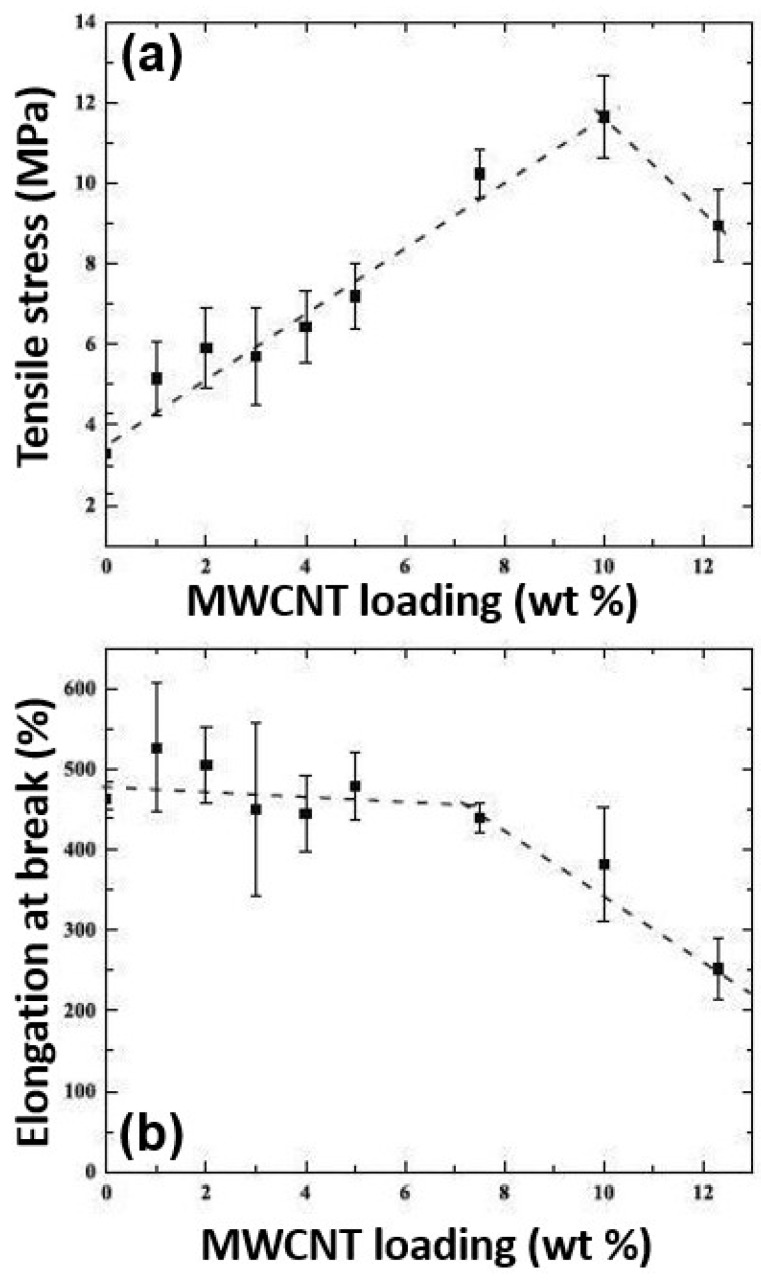
(**a**) Tensile stress at break; (**b**) elongation at break as a function of MWCNT loading. Reproduced with permission from [69], Copyright Elsevier, 2015.

**Figure 7:**
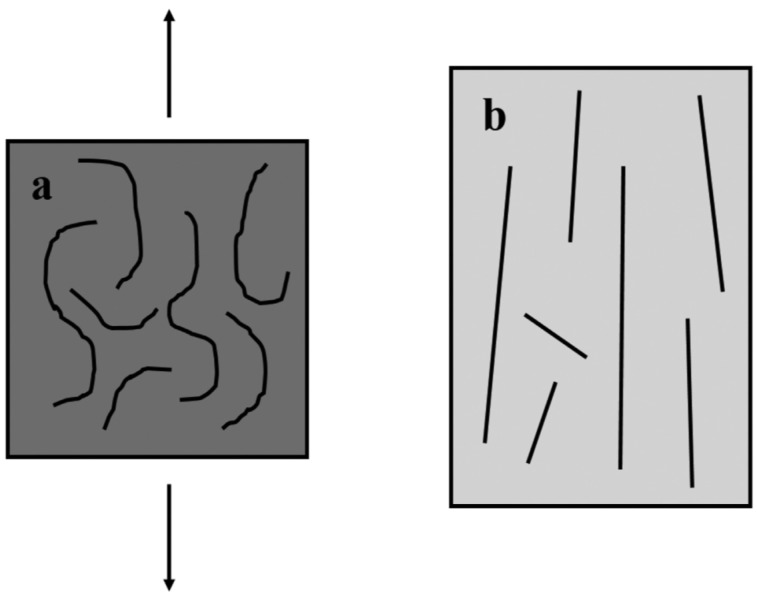
(**a**) Composite sample with coiled MWCNTs before stretching; (**b**) Stretched sample with straight MWCNTs. Reproduced with permission from [69], Copyright Elsevier, 2015.

The composite of SBR loaded with ionic modified MWCNT (10 phr) exhibited impressive enhancements in tensile strength (381% increase) and hardness (34% increase as outcome of the extremely fine dispersion [63]. Falco et al. [66] recorded stress-strain curves at room temperature for SBR compound, SBR/CB composite and SBR/MWCNT composite. It is inferred that nanotubes in the SBR matrix showed remarkable improvement in the tensile strength and strain to failure comparison with the sample of SBR/carbon black composite. The higher aspect ratio of MWCNT and better interface between the two phases composites accounted for such improvements in SBR/MWCNT. Girun et al. [67] observed enhanced mechanical properties in SBR/MWCNT (1 to 10 wt %) nanocomposites fabricated by solvent casting method. Their findings also showed that Young’s modulus of 1 and 10 wt % filled CNTs in SBR compared to SBR without CNTs increased by ~10% and 200%, respectively. The spray drying method followed by subsequent mechanical mixing has been used to prepare styrene-butadiene rubber/carbon nanotubes nanocomposites and studied their mechanical properties [68]. In comparison to pure SBR composites, mechanical properties, such as tensile strength, tear strength, and hardness of the composites filled with CNTs at certain contents were dramatically improved almost by 600%, 250%, and 70%, respectively. Tensile strength of SBR also increased by 21% to 70% for the corresponding CNT contents. Peddini et al. [69] used master batches of discreet well-dispersed MWCNTs in a SBR matrix and subsequently diluted with SBR to prepare sample containing lower MWCNT (1 to 12.3 wt %) loadings and studied its tensile stress-strain behavior. Figure 6 shows the variation of tensile stress at break and elongation at break as a function of MWCNT loading in SBR. It is noted that tensile stress at break increases with increasing MWCNT loading up to 10 wt % and then decrease at higher loadings. This increase reflects the reinforcing effect of filler with good SBR-MWCNT surface bonding between the MWCNT and SBR. A change in response of the elongation at break around 6–8 wt % is also evident. The average elongation at break values for the composites are very close to that of SBR (~450%), up to the loading level of the proposed threshold. It is remarkable to see essentially no decrease in elongation at break with the addition of MWCNT below this threshold. The high elongation at break could be ascribed to a strong interfacial bonding between SBR and tube surface. Alternatively, the possibility of a straightening of the curved or coiled tubes in the stretch direction along with the matrix, as schematically shown in Figure 7, also cannot be ruled out. At higher MWCNT loadings, the elongation at break shows a modest decrease. These workers also investigated mechanical properties of SBS/CB and compared with SBR/MWCNTs composites of similar compositions composites. The corresponding Young’s modulus data at 100%, 200%, and 300% of deformation for these composites revealed no significant influence of CB on SBR on the Young’s Modulus in contrast to SBR/MWCNT composites [70]. On the other hand, an increase in MWCNT content causes a significant increase, mainly at higher strain levels. MWCNT act as reinforcement agents, however this effect does not compromise the strain capability of the elastomer, resulting in materials with higher tenacity in comparison with SBS. Atieh [71] employed MWCNTs functionalized with carboxylic group dispersed it homogeneously in SBR in an attempt to enhance the mechanical properties of these resulting nanocomposites. It was seen that Young’s modulus of the rubber nanocomposites at 10 wt % of MWCNTs loading is ~6 times higher than SBR. Further, tensile strength of nanocomposites also increased due to the compatibility between MWCNTs and SBR. They also extended their work and used amine functionalized MWCNTs to evaluate the extent of reinforcement [72]. The tensile strength of the SBR/functionalized MWCNT (10 wt %) rubber nanocomposites is found to be ~2 (181%) times that of pure SBR. This is ascribed to well-dispersed MWCNT and a good interface between the MWCNT and SBR matrix. An increase of 175% in the Young’s modulus was observed in 10% loaded filer in SBR nanocomposite.

Laoui [74] investigated effect of reinforcing SBR with phenol functionalized carbon nanotubes on the mechanical properties of the resulting nanocomposite. It is noted that the mechanical properties are substantially improved for the small filler loading. The higher increase in tensile strength is also observed at higher MWCNT loadings in SBR. Stress-strain curve also showed a strength of the SBR/MWCNT (10 wt %) that is almost three times (282%) than that of pure SBR, while the strain is decreased from 21 to 7 due to dispersion of CNTs and a good interfacial bond between the functionalized CNT and SBR matrix. It is also noted that the Young’s modulus increases by 40% and 240%, corresponding to 1 and 10 wt % of functionalized MWCNT. Das et al. [78] used the mixing method to incorporate MWCNTs in a rubber blend in a 50:50 blend of solution-styrene-butadiene rubber and butadiene rubber. It is noted that stress increases with the incorporation of the CNTs with a sharp rise of the initial Young’s modulus.

**Figure 8:**
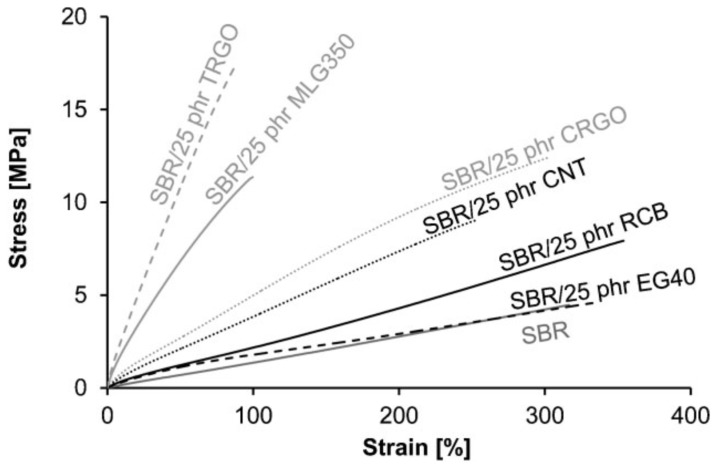
Stress-strain curves of SBR with 25 phr of different carbon fillers. Reproduced with permission from [79], Copyright Wiley, 2014.

**Figure 9:**
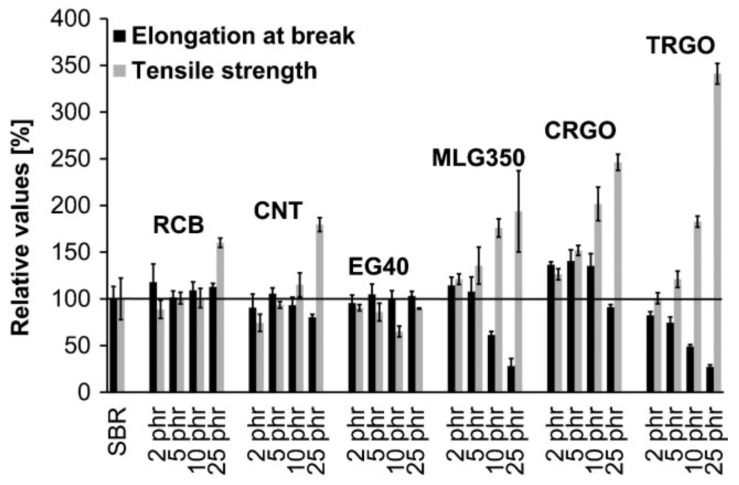
Elongation at break and tensile strength for SBR/C filler composites relative to neat SBR. Reproduced with permission from [79], Copyright Wiley, 2014.

**Figure 10:**
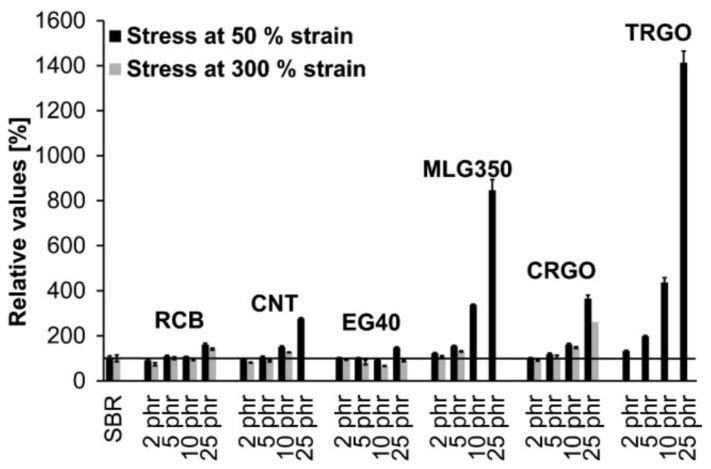
Stress at 50% and 300% strain of styrene butadiene rubber (SBR)/C based fillers as relative values compared to neat SBR. Reproduced with permission from [79], Copyright Wiley, 2014.

Schopp et al. [79] prepared SBR nanocomposites containing different carbon-based fillers using an aqueous dispersion blend technique and carried out stress/strain tests of these SBR nanocomposites, as displayed in Figure 8. The elongation at break and tensile strength for SBR/C (2, 5, 10, 25 phr) filler composites relative to neat SBR are displayed in Figure 9. It is noted that addition of all these carbon fillers affords higher tensile strength, which increases with an increasing filler content. Carbon filler performance improves with the following filler ranking: Expanded graphite < Rubber carbon black < CNT < Multilayer Graphene < Chemically reduced graphite oxide < Thermally reduced graphite oxide. The highest tensile strength increases (240%) is found for SBR/25 phr TRGO. Among the carbon fillers, only the CRGO addition simultaneously increases elongation at break and tensile strength. Figure 10 show stress at 50% and 300% strain of SBR/C based fillers as relative values as compared to neat SBR. This clearly reveals that all fillers show increasing filler content in SBR increase tensile stress. It is noted highest stress at 300% strain is observed for SBR containing 25 phr CRGO (260%). Das et al. [73] reported solution styrene butadiene rubber composites reinforced with EG, grapheme nanoplatelets and MWCNTs. It was concluded that SBR/MWCNT showed significant improvement in mechanical properties when compared to other composites. The high aspect ratio of MWCNT enabled forming a network at low filler loading, and, consequently, good reinforcement effect was observed.

The formation of hybrid fillers (MWCNT and EG) showed improvements in mechanical properties due to the synergistic effect. The hardness of SBR composites filled with graphene showed sharp increase in hardness and modulus at 300% elongation [77]. Bhowmick et al. [81] filled SBR with modified and unmodified carbon nanofiber and expanded graphite. The comparison of mechanical properties of their nanocomposites as compared to the gum on the basis of illustrates that carbon nanofiber increased the modulus by 101%, while tear strength increased by 79% at 6 phr loading. On modification, the carbon nanofiber showed 150% increment in modulus and 113% in tensile strength of the nanocomposite, over the gum control. Xing et al. [83] fabricated graphene/SBR nanocomposites by a modified latex compounding method and measured tensile strength and strain at break and strength at 300% strain. It is noted that a 260% increase in the tensile strength and a 140% improvement in the strain at break are achieved even at the graphene loading as low as 0.3 phr. At 7 phr of GE in SBR, the tensile strength of the nanocomposite increased to ~11 times higher than that of SBR. For the same time, the strain at break of the nanocomposite remains the same as that of pure SBR. Mechanical properties of expanded graphite and modified expanded graphite filled SBR/BR nanocomposites were also investigated [85].

Several other studies are reported on investigating the mechanical properties of SBR filled with hybrid fillers. Organically functionalized MWCNTs (O-MWCNTs) showed improved mechanical properties of NR/SBR composites [55]. These findings showed that elongation at the break of NR/SBR composites filled with 1.5 phr O-MWCNTs under optimized conditions was found to be 450% as compared to 376% for pristine NR/SBR composites. Nanocomposites based on NR/EPDM/MWCNT were also prepared in an internal and a two roll-mill mixer in two steps and effect of MWCNTs and studied for their mechanical properties [56].

Chen et al. [190] used graphene oxide exhibiting various oxidation degrees and studied its reinforcing performance in SBR. Tensile strength and tear strength of SBR/GO nanocomposites increased by 271.3% and 112.3%, respectively compared with neat SBR. The tensile strength and tear strength of ionic liquid functionalized GO (GO-IL)/SBR nanocomposites with 5 phr of GO-IL increased by 505 and 362%, respectively, as compared with neat SBR [191]. SBR/3D segregated graphene (IL-3DGE) dramatically enhanced its mechanical properties [192]. The incorporation of 1.66 vol % IL-3DGE significantly increased the tensile strength by 516% compared to neat SBR. The excellent properties of the composites were attributed to the strong interfacial interactions. GO has also been used as reinforcing filler in immiscible XNBR/SBR blends [193]. The incorporation of only 0.3 phr GO as a reinforcing filler significantly improved the tensile strength (71%) and tear strength (94%) of XNBR/SBR blend. XNBR/GO nanocomposites that were fabricated by aqueous phase mixing of GO colloidal dispersion with SBR latex and a small loading of XNBR latex, followed by coagulation were thoroughly investigated for mechanical properties [194]. The results showed an enhancement in the mechanical strength of nanocomposites with the increase of oxidation degree of GO. The tensile and tear strengths of SBR/XNBR/GO filled with 3 phr GO as compared to neat blend increased by 255.3% and 141.5%, respectively. SBR filled with 5 phr of PVP modified graphene oxide (PGO) significantly improved tensile strength and tear strength, respectively [206]. It was suggested that PVP molecules could have strong interaction with GO via hydrogen bond and account for this.

Zhang et al. [87] reported the tensile curves of SBR/CB and SBR/CB/RG composites fabricated via two-roll mill mixing and corresponding mechanical data is represented in Table 7. SBR/CB (100/10 and 100/13 phr) blends showed limited improvements on the tensile properties were observed in contrast to SBR/CB/reduced grapheme (RG) (100/10/1, 100/10/2,100/2/3 phr) composites exhibiting significant improvements. The moduli at 200% elongation (M200) of the SBR/CB-RG blends were found to be higher than those of the SBR/CB blends at no expense of elongation. The elongation at break-even increased from 260% to 300% when 1 phr of RG was applied compared to the SBR/CB blend filled with 10 phr CB. The tensile strength of the blends was enhanced from 3.5 to 4.9 MPa, increased as much as 40% after addition of 1 phr RG. The enhancement of the tensile properties after the application of CB-RG hybrid filler compared to single CB filler can be attributed to the better reinforcement effect of RG on the SBR matrix. At higher loadings of RG (2 and 3 phr), M200 of the SBR/CB-RG composites increased without pronounced improvements on tensile strength and elongation at break. In another study, the introduction of thermally reduced graphene (0.25 phr) to SBR/MWCNT (1 phr) increased the tensile strength of the resulting composites by 49.5% as compared to SBR/MWCNT (1 phr) [88]. Such an improvement in mechanical properties was ascribed to the synergistic dispersion between thermally reduced graphene and MWCNTs and the strong interfacial interaction between the hybrid fillers and the rubber matrix. However, further addition of thermally reduced graphene (0.5 phr) reduced the tensile strength of the SBR/MWCNT/composites due to the formation of agglomerates of graphene. Tang et al. [89] used a continuous 3D hybrid (HG) consisting of halloysite tubular clay, HNT (referred as H), and tannic acid functionalized graphene, TAG (referred as G) as reinforcing filler in SBR and studied their mechanical properties. It is noted that HG ternary composites exhibit relatively much larger Young’s modulus, tensile strength, and tear strength when compared to SBR-H and SBR-G binary composites. This is in all probability due to the extraordinary synergistic effects of halloysite tubular clay and functionalized graphene. The combination of hydroxyl-functionalized exfoliated montmorillonite (Fe-MMT) and cetyltrimethylammoniumbromide-modified MWNT hybrids has been used to prepare SBR composites by the solution method [90]. Remarkable improvement in modulus (stress at the strain of 100%) and tensile strength at low loadings were observed due to homogenous dispersion of the hybrid nanofillers in the SBR matrix. Bhuyan et al. [207] measured mechanical properties of MWCNT/hectorite hybrid (HMH) hybrid filler reinforced styrene SBR. These findings show significant improvement in tensile strength (210%) and elongation at break (42%) of SBR/HMH nanocomposite at 0.7 wt % HMH. Such superior reinforcing of hybrid filler compared to individual fillers is ascribed to synergistic effect. Alternatively, extraordinary improvement in mechanical properties at such low filler contents could also be attributed to the enhanced level of MWCNTs dispersion in SBR matrix due to the simultaneous presence of hectorite layers.

### 3.3. Mechanical Properties of Carbon Filler Incorporated NBR and Its Blend Nanocomposites

**Figure 11:**
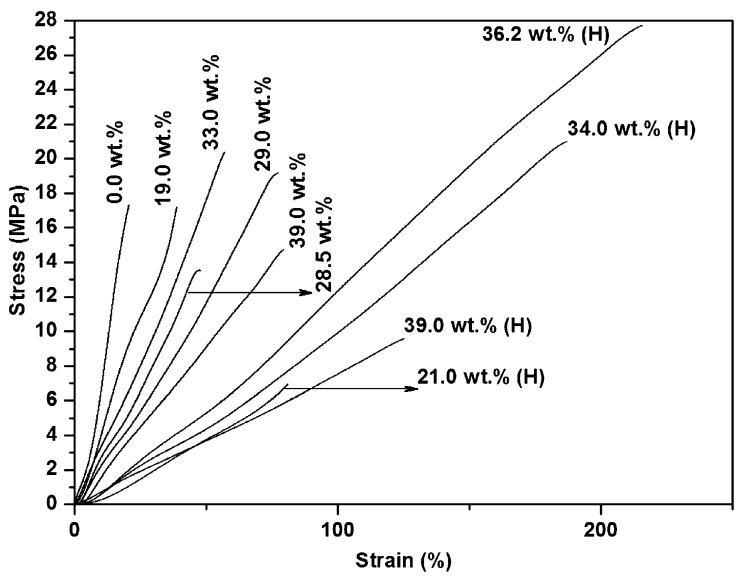
Characterization of mechanical properties-tensile properties (stress vs. strain) of (H)NBR/MWCNT nanocomposites with different AN contents prepared by MC. Reproduced with permission from [100], Copyright Elsevier, 2010.

NBR is highly resistance to the hydrocarbon oil that is required in fuel hoses, o-rings, and gaskets. However, it is susceptible to ultraviolet light and ozone attack and has been overcome by hydrogenating to HNBR [17]. These high performance rubbers exhibit excellent abrasion/ adhesion resistance and superior mechanical properties for their wide range of applications. NBR is not crystallizable under high strain, and therefore the reinforcing fillers are generally incorporated to yield sufficiently high mechanical properties [91,92,93,94,95,96,97,98,99,100,101,102,103,104,105,106,107,108,109,110,111,112,113,114,192,193,194,195,196]. Chougule and Giese [93] reported significant mechanical reinforcing effects of CNT as compared with CB in NBR due to good dispersion and effective interaction. The influence of ACN content on the mechanical properties of NBR/MWCNT nanocomposites has been investigated [95]. The lower volume fraction of MWCNTs produced a significant increase in tensile strength and energy at break when compared with carbon black due to high degree of dispersion. In another work, the mechanical properties of nitrile rubber reinforced with 0 to 15 phr of MWCNT, conductive (CB, CB), and precipitated silica prepared by the two roll mill method and corresponding data are recorded in Table 8 [97]. It is clearly seen that tensile strength, modulus (100%), and hardness enhanced with the increasing of the loading of filler. Interestingly, MWCNT gives the highest level of reinforcement when compared to other conventional reinforcing fillers. However, except for the MWCNT filled system, the elongation at break appears to increase with increasing filler loading. Such an increase is believed to be due to the slippage of non-cross-linked rubber molecules around filler particles which increases the specimen volume under high extension. When compared to the unfilled system, the abrasion loss of the filled systems decreased and the heat buildup values decrease with increasing filler loading, Ryu et al. [98] studied stress-strain curves of NBR (matrix) and its composites filled with CNT and CB. This study showed that tensile strength and tensile modulus increase with an increase of CNT content when compared with the matrix and rubbers. Tensile strength and tensile modulus of the composite on adding 9 phr of CNT increased to 31% and 91%, respectively. Likozar and Blaz [100] investigated the effects of acrylonitrile content on both properties of NBR. Figure 11 shows the typical tensile stress–strain curves of the vulcanizates (NBR and HNBR of 0.0–39.0 wt % of acrylonitrile content) filled with 30 phr of MWCNT. It was concluded that tensile strength of HNBR nanocomposites increases substantially with acrylonitrile content in the range 21.0–36.2 wt %. Also, the stress at small strain (under 20%) increases more remarkably with increasing acrylonitrile content, when compared to NBR/MWCNT composites. It is also inferred that HNBR/MWCNT (36.2 wt % AN content) nanocomposite exhibits highest tensile strength and elongation at break when compared to those of other reinforced composites due to the high aspect ratio and large surface areas of carbon nanotubes. MWCNTs (1, 3, and 5 wt %)-filled thermoplastic polyurethane-urea (TPU)/XNBR blend nanocomposites showed significantly improved mechanical properties as compared to the neat blend [110]. It was inferred that the addition of 5 wt % CNTs in XNBR:TPUU blend increased the tensile modulus (45 MPa) and tensile strength at break (33 MPa) when compared to the respective values of 9.9 MPa and 25 MPa for the neat XNBR:TPUU blend. In all probability, well-dispersed CNTs in the blend effectively absorb the applied stress and improve the mechanical stiffness in XNBR:TPUU/CNT nanocomposites. Salehi and coworkers [107] observed that tensile strength, elongation at break, stress at different elongation, and hardness of pure NBR are enhanced on adding MWCNT or silica as reinforcing fillers. The mechanical properties of the composites are further enhanced in the presence of combination of silica and CNT fillers in NBR. Thus, the tensile strength increases to 71% on adding 3 phr CNT to the NBR sample containing 25 phr silica when compared to the tensile strength value of the silica (25 phr) filled NBR due to the synergistic effect.

NBR/EG (5, 10 phr) nanocomposites were prepared by the mechanical blending (microcomposites) and latex compounding technique (nanocomposites) and their mechanical properties [101]. These findings reflected superior tensile properties at the same graphite loading of nanocomposites to that of micro-composites. This suggested that nano-size dispersed graphite can be dispersed more uniformly and reinforce rubber more effectively than micro-size graphite. Liu and coworkers [102] investigated the variation of tensile strength and elongation at break with EG content in the NBR/EG nanocomposites. It was noted that the tensile strength increased by 78% for those nanocomposites containing only 5 phr expanded graphite. They believed that the nanoscale dispersion of the EG nano sheets within NBR matrix account for such enhancement. The drop in tensile strength of the composites at higher loadings was probably due to the aggregation of the EG. The elongation at break decreased only slightly with increasing EG content. Mechanical properties of NBR/EG/CB nanocomposites with different graphite (1, 3, 5 phr) show that elongation at break rises significantly, even for a small increase in the amount of graphite [103]. Interestingly, tensile stress (Young’s modulus) at 100% elongation is the highest, corresponding to 1 phr graphite in the composites. It is believed that, at very low loading amount, graphite could be dispersed better in the matrix. Further, it was noted that hardness and tear strength of the NBR/EG/CB nanocomposites increase only to a very small extent.

**Figure 12:**
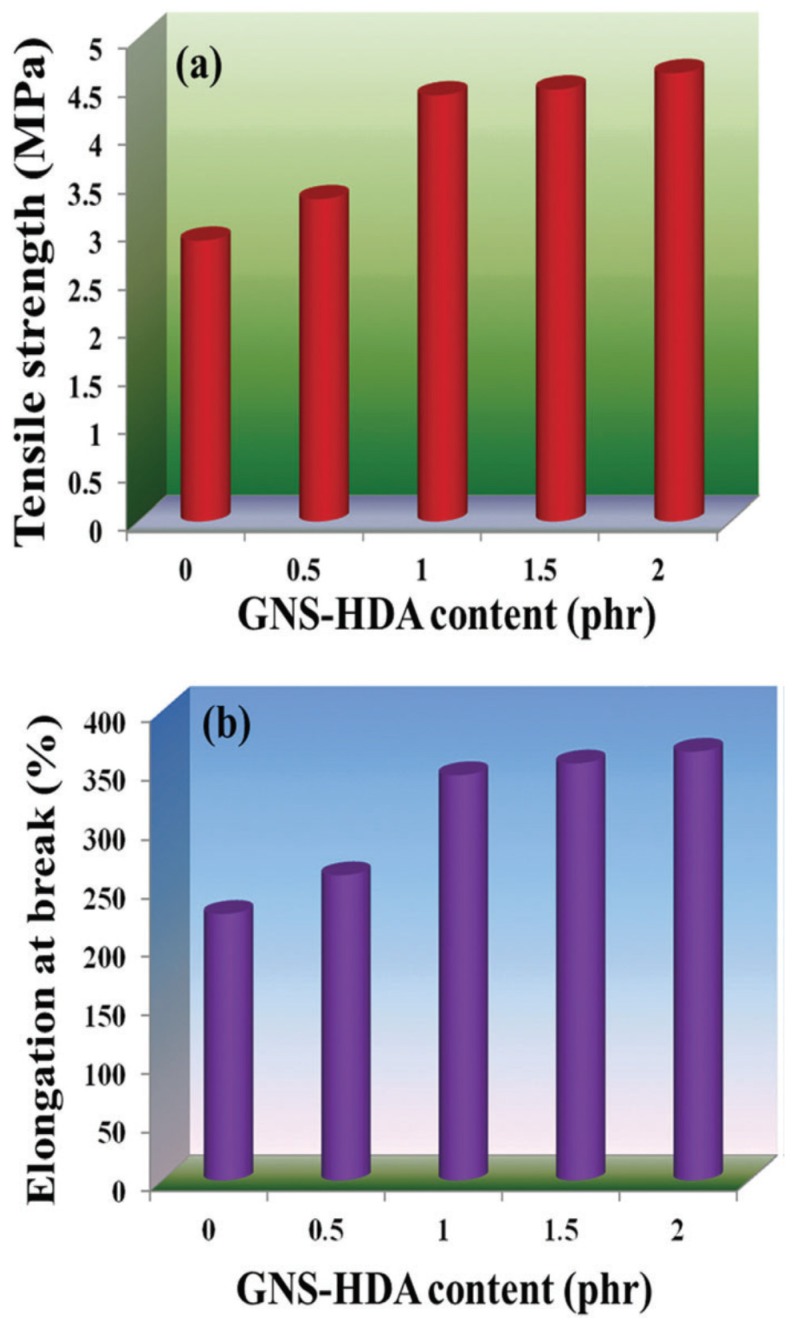
(**a**) Tensile strength and (**b**) elongation at break of neat carboxylated acrylonitrile butadiene rubber (XNBR) and various hexadecyl amine functionalized graphene (GNS-HDA)/XNBR composites. Reproduced with permission from [104], Copyright RSC, 2017.

**Figure 13:**
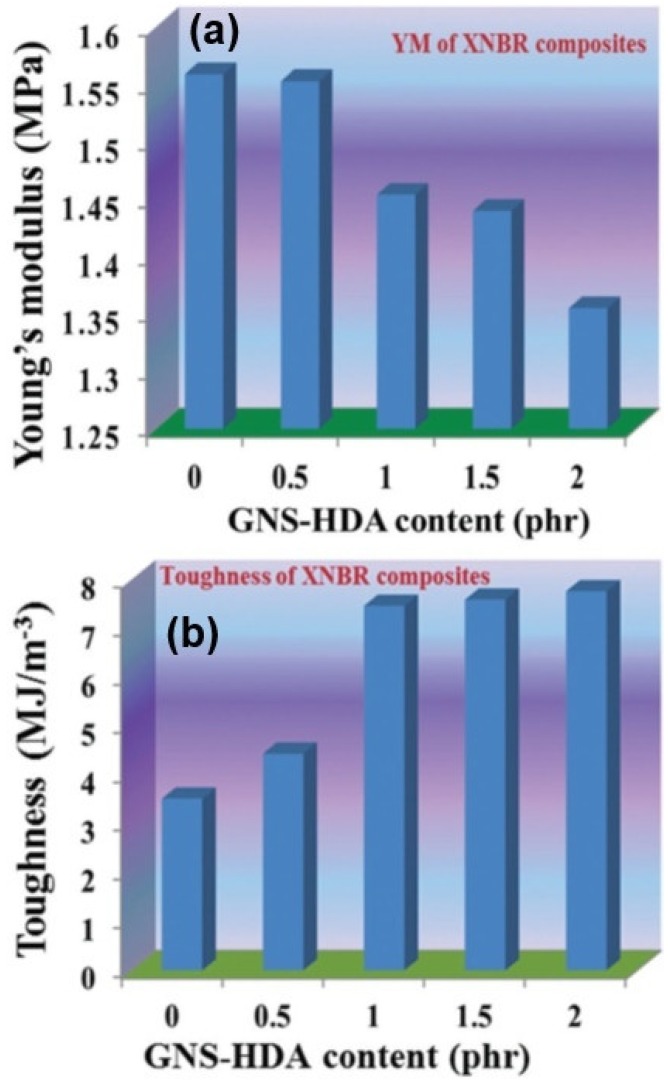
(**a**) Young’s modulus of neat XNBR and various GNS-HDA/XNBR composites; (**b**) Toughness of neat XNBR and various GNS-HDA/XNBR composites. Reproduced with permission from [104], Copyright RSC, 2017.

Manna and Srivastava [104] fabricated hexadecyl amine functionalized graphene (GNS-HDA) filled carboxylated nitrile rubber nanocomposites as flexible dielectric materials. The variation of tensile strength, elongation at break, and toughness of neat XNBR and GNS-HDA/XNBR are displayed in Figure 12 and Figure 13. It is noted that XNBR filled with 2 phr of GNS- exhibited a significant improvement in tensile strength (60%) and elongation at break (62%) when compared to neat XNBR. The toughness of the GNS-HDA/XNBR composites also increases significantly with filler loading. Such an enhancement in the mechanical properties of XNBR/GNS-HAD nanocomposites could be attributed to the interfacial interaction between GNS-HDA and the XNBR matrix and effective load transfer from the filler to the polymer matrix. Alternatively, the role of molecular level dispersion of GNS-HDA and its wrinkled shape leading to mechanical interlocking and transmitting the applied stress to XNBR also cannot be ruled out. It is inferred also that the Young’s modulus of the composites is remarkably reduced (13%) in 2 phr of GNS-HDA loaded XNBR. This clearly indicates the reduced stiffness and enhanced flexibility of fabricated XNBR/GNS-HDA nanocomposites. The mechanical properties of graphene filled NBR rubber nanocomposites have also been reported by other workers [105,106]. The tensile modulus NBR-reduced grapheme oxide (rGO) composites prepared by solution mixing method at a 0.1-phr rGO loading greatly increased above 83%, 114%, and 116% at strain levels of 50%, 100%, and 200%, respectively compared to the 0.1-phr GO loaded sample [195]. The observed enhancement was highly attributed to a homogeneous dispersion of rGO within the NBR matrix. RGO/HNBR composites exhibits enhanced mechanical properties compared with HNBR [196].

Thermoplastic polyurethane (TPU) with NBR finds appreciations in the field of automotive gaskets, gaskets/co-extrusion, protective covers, tubing pipes, and grips, etc. [12]. The presence of TPU in the blend accounts for the improvement in tensile strength, fuel/oil, weather, ozone and oxygen resistance, and NBR promotes the solvent resistance and thermal stability. Desai et al. [109] observed co-continuous phase in TPU:NBR (50:50) blend, owing to uniformly dispersed phases. However, there exists limited work only on the carbon based hybrid fillers reinforced Polyamide 6/NBR/SWCNT [91]. NBR/PVC/SWCNT, [92] TPPU/XNBR/MWCNT, [110] EPDM/NBR/MWCNT [111], and hybrid filled TPU/NBR blend nanocomposites [112,113,114] MWCNTs filled thermoplastic polyurethane-urea (TPUU) and carboxylated acrylonitrile butadiene rubber (XNBR) blend nanocomposites exhibited increased tensile modulus from about 9.90 to 45.3 MPa, at 3 wt % loading of CNT [110]. Hoikkanen et al. [111] prepared MWCNT/NBR/EPDM blends nanocomposites by melt mixing methods and studied their mechanical properties. They concluded that the mechanical properties of these blend nanocomposites are controlled by the degree of dispersion of the nanotubes.

**Figure 14:**
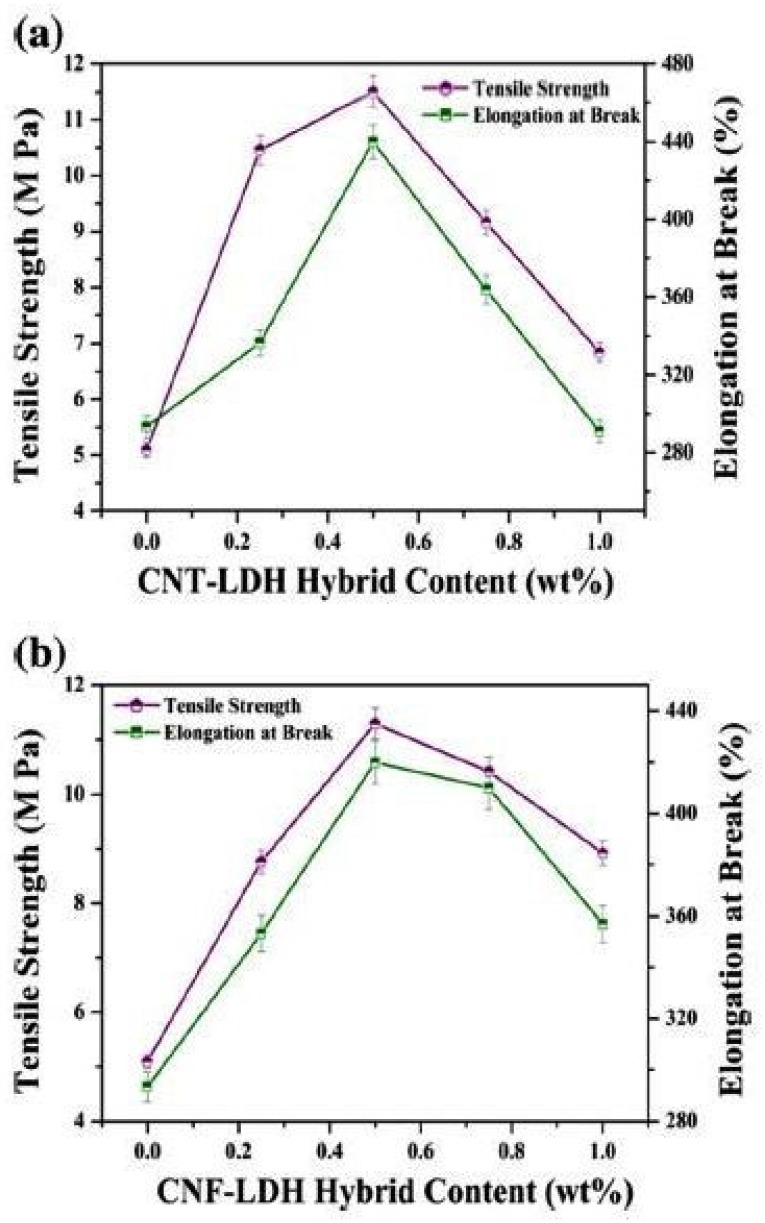
(**a**) Variation of tensile strength and elongation at break of TPU/NBR (TN) nanocomposites with Zn-LDH/surfactant modified CNT-layered double hydroxide (SFCNT-LDH) hybrid content; (**b**) Variation of tensile strength and elongation at break of TN nanocomposites with SFCNFLDH hybrid content. Reproduced with permission from [112], Copyright Springer, 2016.

**Figure 15:**
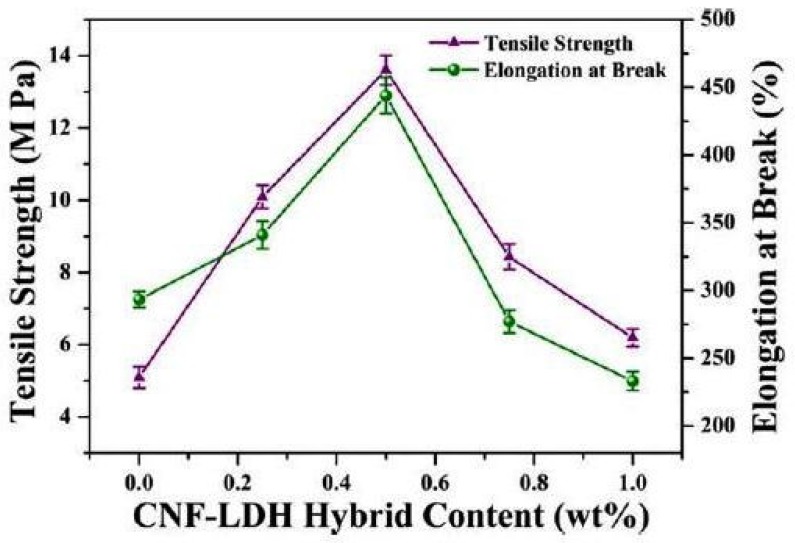
Variation of the tensile strength and elongation at break of the TN nanocomposites versus the SFCNF-LDH hybrid content. Reproduced with permission from [113], Copyright Wiley, 2016.

**Scheme 2:**
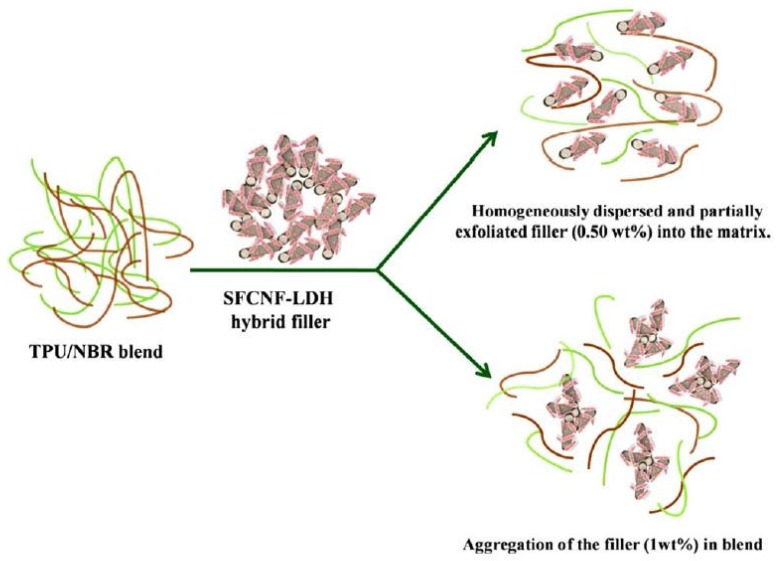
TN blend without and with the filler to explain the mechanical properties. Reproduced with permission from [113], Copyright Wiley, 2016.

Srivastava and his group [112] reported the variation of tensile strength (TS) and elongation at break (EB) of TPU/NBR (referred as TN) blends nanocomposites with respect to SFCNT-LDH and SFCNF-LDH hybrid filler content in Figure 14. It is also noted that 0.50 wt % SFCNT-LDH loaded TN nanocomposites exhibit improvement in tensile strength (126%) and EB (1.50 times) compared to neat TN. On the other hand, with respect to neat TN, 0.50 wt % SFCNF-LDH hybrid filled TN nanocomposites also show 122%- and 1.43-times improvements in tensile strength and EB, respectively. The enhanced mechanical properties of TN nanocomposites clearly suggest the reinforcing effect of both the SFCNTLDH and SFCNF-LDH hybrid fillers in TN. They ascribed such superior mechanical properties of TN due to the synergistic effect of SFCNT (or SFCNF) and LDH. Further, at higher filler loadings, tensile strength and elongation at break slightly decrease due to the tendency of the hybrid fillers to agglomerate, giving rise to initiating sites for crack propagation. In another work, carbon nanofiber (CNF)-layered double hydroxide (LDH) hybrid through a noncovalent assembly using sodium dodecyl sulfate as bridging linker between magnesium–aluminum LDH and CNF were used as nanofiller in TPU/NBR (50:50) blend [113]. Figure 15 shows the variation of the tensile strength and elongation at break of the TN nanocomposites with respect to the SFCNF–LDH hybrid filler. The enhancement in the mechanical properties clearly indicated the reinforcing effect of SFCNF-LDH in the TN matrix. It was also noted that the mechanical measurements of 0.50 wt % hybrid loaded TN blend exhibited maximum improvements in the elongation at break and tensile strength of 1.51 times and 167%, respectively. Their findings also confirmed the synergistic effect of SFCNF and LDH in the mechanical properties of the TN nanocomposites. Scheme 2 was proposed to explain the mechanical properties of TN blend in the presence of the SFCNF-LDH hybrid filler, Srivastava and workers [114] also applied their approach to assemble MgAl layered double hydroxide onto pristine carbon nanotubes using sodium dodecylsulfate and used as hybrid nanofiller in the development of high-performance TPU/NBR (1:1 *w*/*w*) blend nanocomposites. The tensile strengths of TPU/NBR filled with 0, 0.25, 0.50, 0.75, and 1 wt % SFCNT-LDH hybrids are 5.09, 11.7, 13.8, 10.6, and 9.78 MPa, respectively. The respective elongation at break values is 293%, 530%, 513%, 436%, and 413%. It is observed from the data that the 0.50 wt % SFCNT-LDH hybrid loaded TPU/NBR exhibits the maximum enhancement in tensile strength (171%) and the elongation at break (1.8 times) as compared to pure TPU/NBR due to the optimum dispersion of SFCNT-LDH hybrid filler that causes enhanced interaction between the matrix and nanofiller.

### 3.4. Mechanical Properties of Carbon Filler Incorporated SR and Its Blend Nanocomposites

SR are one of the most important functional polymers, which have received considerable interest owing to their unique properties, e.g., excellent physical, chemical, and thermal stability, low glass transition temperature, clarity; biocompatibility, nonreactivity; and, low surface energy [18]. Poly(dimethylsiloxane), also referred as PDMS, is a silicon-based organic polymer that is composed of a repeating [SiO(CH_3_)_2_] unit and exists in rubber state at room temperature, as its glass transition temperature is less than −120 °C. It is useful in casting moulds, micro-fluidic devices, the automotive and aerospace industry, cables for appliances and telecommunications, cooking, baking and food storage products, medical implants, in electrical insulation products, etc. Despite several advantages, the vulcanized neat silicone rubbers usually have poor mechanical properties and low thermal/electrical conductivity restricting its use in many industrial applications. Therefore, silicon rubber filled with carbon based nanofillers have been reported [115,116,117,118,119,120,121,122,123,124,125,126,127,128,129,130,131,132,133,134,135,136,137,138,139,140,141,142,143,197,198,199,200]. Room temperature vulcanized (RTV) vulcanizates on adding 2 phr of CNTs increased the Young’s modulus by 272% and reached as high as ~706% at 8 phr [116]. In another work, vulcanized (RTV) silicone rubber/MWCNT nanocomposites have been prepared at room temperature [117]. It is noticed that addition of 5 phr of MWCNTs in RTV imparted good comprehensive performance of the composite as indicated by enhanced tensile strength, tear strength, elongation at break.

Shang et al. [118] fabricated a series of high temperature vulcanized silicone rubber (HTVSR)/MWCNTs nanocomposites with different CNT contents. In this, MWCNTs were pretreated by the chitosan salt before being incorporated into the HTVSR. Table 9 shows that tensile properties of the HTVSR nanocomposites are significantly increased due to the uniform dispersion of chitosan salt pretreated MWCNTs in HTVSR matrix. It is noted that tensile stress, elongation at break, and modulus increased with the increasing MWCNTs (below 8 wt %) contents in HTVSR. At further higher filler loading (11 wt % of MWCNT), tensile stress and modulus still increased while the elongation at break decreased, which meant that more MWCNTs made the HTVSR more brittle but tougher. The increasing of the tensile strength of the nanocomposites indicated that chitosan salt treated MWCNTs and HTVSR had very strong interfacial adhesions, and the MWCNTs helped to transfer some tensile force when the HTVSR/MWCNTs nanocomposite was stretched.SR filled with ball mill prepared hybrid filler consisting of 2.5 phr CB and 1.0 phr MWCNT shows improvement in tensile strength and strain to failure due to the good dispersion and synergistic effects of MWNT and CB [119].

Cha et al. [122] studied effect of incorporating individual MWCNTs and continuous MWCNT bucky paper on the mechanical properties of the PDMS composite films. The tensile strengths of the composite films filled with the bucky paper and as-received MWCNTs were improved by 2268% and 531% when compared to that of the pure PDMS film. The tensile strength of the composite film was found to be superior that obtained by filling with a commercially available bucky paper fabricated under the same processing. Wu et al. [123] investigated the mechanical properties of PDMS and its MWCNT filled nanocomposites. The elastic modulus of pure PDMS (1.65 MPa) was increased in 1.0 wt %, 2.0 wt %, and 4.0 wt % CNT loaded in PDMS to 1.71, 1.91, and 2.34 MPa, respectively. The good bonding between CNTs (4 wt %) and PDMS, as evident from fracture surface analysis, account for this. Katihabwa et al. [125] also studied mechanical properties MWCNTs reinforced SR nanocomposites prepared through a high-shear mechanical mixing technique using DCP as a curing agent. The tensile stress of the SR increased with CNT contents and became four times higher for 20% filler content due to well-dispersed CNTs in the rubber matrix. In contrast, the elongation at break of the nanocomposites decreases linearly with the increase of CNT content, probably due to poor filler-polymer interaction.

Mazlan et al. [127] reported effects of ultrasonic and mini extruder compounding processing techniques on modulus at 100% elongation and elongation at break (%) of SR filled with 0.5, 1.0, and 1.5 vol % MWCNT. These findings clearly showed the reinforcing effect of MWCNT in the PDMS, as evident from the increase in MWCNT/PDMS (1.5 vol %) corresponding to 1.4785 MPa as compared to PDMS (0.6735 MPa). The elongation at break in MWCNT/PDMS (1.5 vol %) also increased to 140 MPa with respect to neat PDMS (129.6%). The mechanical properties are also improved in MWCNT/PDMS nanocomposites that were prepared by mini extruder compounding process due to the good dispersibility of the tubes in the silicone matrix. Yadav et al. [128] investigated mechanical properties of PDMS/MWCNT (covalently functionalized) nanocomposites synthesized via nitrene chemistry and observed larger tensile modulus and tensile strength of all the nanocomposites compared to the neat PDMS elastomer. Such enhancements in mechanical properties of the nanocomposites are attributed to the homogeneous dispersion of silicone-g-MWCNTs in the silicone elastomer matrix. In addition, graphene [135,136,137,139,140,197] and graphene oxide [198,199,200] has been incorporated to enhance the mechanical properties of the silicone rubber. Mechanical property measurements of liquid silicone rubber/GO functionalized with triethoxyvinylsilane composites (0.3 wt %) displayed a 2.3-fold increase in tensile strength, 2.79-fold enhancement in tear strength, and 1.97-fold reinforcement in shear strength when compared with the neat liquid silicone rubber [199]. Gan et al. [198] studied effect of vinyl concentration of the silicone rubber on the mechanical properties of the SR/GO composites. It was found that the uniformly dispersed GO sheets within the SR matrix increase the mechanical properties of the SR. The 3-aminopropyltriethoxysilane (APTES) graphite oxide (3.0 wt %) reinforced PDMS showed ~71% enhancement of Young’s modulus [200].

**Figure 16:**
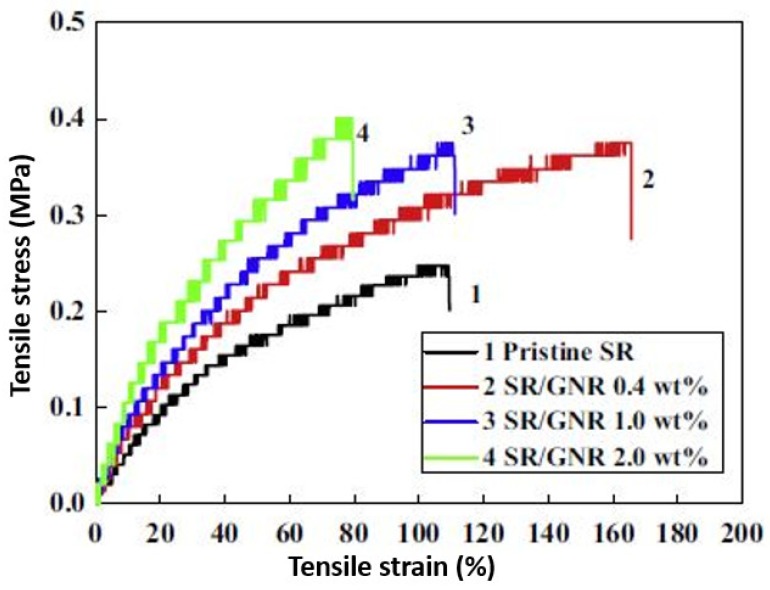
Typical stress-strain behaviors of the pristine silicone rubber (SR) and the SR/graphene nanoribbon (GNR) nanocomposites. Reproduced with permission from [135], Copyright Elsevier, 2015.

The study of Bai et al. [120] on tensile testing of SR/RGO nanocomposites revealed that an improvement in mechanical properties enhanced with increasing the reduction degree of GO simultaneously. The vulcanized SR/chemically reduced graphene, rGE (30 wt %)/SiO_2_ on adding (rGE)/SiO_2_ (3 wt %) show a tensile strength of 6.13 MPa (up to 25 times), tear strength of 18.08 KN/m, and elongation at break of 267%, several times higher than those of rGE/SR nanocomposite [197]. Functionalized graphene (FG) room-temperature-vulcanized silicone rubber (RTVSR) nanocomposites were also prepared by in-situ reduction of graphene oxide [132]. This was noted that 0.5 wt % loading in RTVSR led to maximum improvement in tensile strength (175%) compared to neat RTVSR. This is ascribed to the homogeneous dispersion of FG in the matrix silicone rubber and strong interfacial adhesion between FG/RTVSR ensuring efficient load transfer at the interface. Similarly, elongation at break was also enhanced in 0.5 wt % FG filled RTV by 67% higher when compared to neat RTVSR. Zong and coworkers [133] prepared functionalized graphene (FG) by a reduction of graphite oxide by hydrazine hydrate and subsequently used in development of silicone rubber nanocomposites. These silicone rubber nanocomposites exhibited significant improvements in tensile strength (198.3%) and elongation at break (268.2%) as compared to neat silicone rubber. It was found that the tensile stress and Young’s modulus of neat SR increased on adding 2.0 wt % of graphene by 67% and 93%, respectively. Interestingly, tensile strain of SR increased up to 1.0 wt % filled SR and then declined. Mechanical properties of graphene nanoribbon (GNR) incorporated SR were significantly enhanced [135]. Figure 16 shows typical stress-strain behaviors of the pristine SR and SR/GNR nanocomposites. It is inferred that tensile stress and Young’s modulus of SR filled with 2.0 wt % of GNR content enhanced by 67% and 93% respectively. In addition, elongation at break of the SR/GNR (0.4 wt %) increased by 64% compared to SR. When the GNR amount reached to 2.0 wt %, although the tensile strain decreased to some extent due to stronger molecular interactions between the SR and the GNR, the tensile stress and Young’s modulus increased by 67% and 93% respectively. Such improvements in the mechanical properties of SR nanocomposites are ascribed to the good dispersion of the GNR and good interfacial interactions between the GNR and the SR.

**Figure 17:**
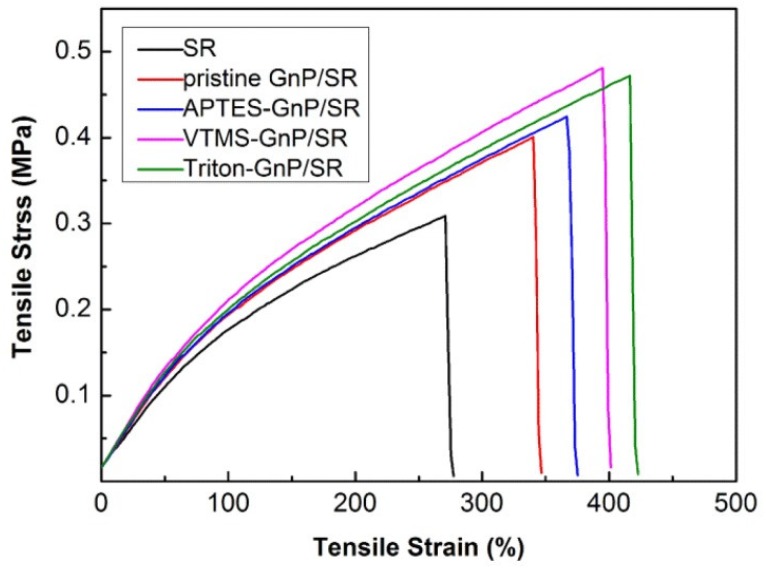
Typical stress-strain curves of neat silicone rubber (SR) and the composites filled with various graphene platelets (GNP). Reproduced with permission from [137], Copyright Wiley, 2016.

**Figure 18:**
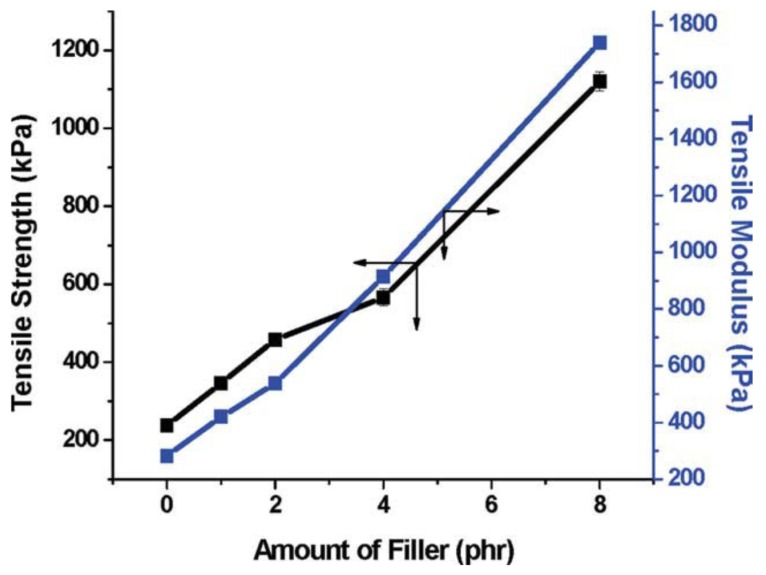
Plot of tensile strength and tensile modulus for in situ prepared amine modified CNF-hydroxyl polydimethylsiloxane (PDMS) nanocomposites as a function of filler loading [138].

Zhang et al. [137] used graphene nanoplatelets that were functionalized by aminopropyltriethoxysilane (APTES), vinyltrimethoxysilane (VTMS), and Triton X-100 and subsequently used as nanofillers in SR. Figure 17 shows considerable improvement in the mechanical properties of SR after incorporating surface modified graphene nanoplatelets. The silane treated graphene-based SR composites showed superior mechanical properties. It was observed that composites reinforced with modified grapheme nanoplatelets showed better tensile strength and elongation at break when compared with the pristine graphene nanoplatelets/SR composite. The mechanical properties of the VTMS-graphene nanoplatelets based composite were found to be superior to that of the APTES treated counterpart. They ascribed it to the stronger interfacial interactions between VTMS-graphene nanoplatelets and silicone chain resulting from the formation of chemical bonds. Roy and Bhowmick [138] prepared polydimethylsiloxane (PDMS)-amine modified CNF based nanocomposites by in-situ and conventional ex-situ methods. Figure 18 shows a combined plot of tensile strength and tensile modulus with increasing filler concentration for in situ prepared amine modified CNF/hydroxyl PDMS nanocomposites. It is noted that the tensile strength increases by 45%, 92%, 137%, and 370%, while tensile modulus enhanced by 48%, 90%, 223%, and 515%, corresponding to 1, 2, 4, and 8 phr filler loadings, respectively. They suggested that mechanical properties thrive at their maximum for this filler–matrix combination since dispersion is improved by chemical functionalization.

Hu et al. [139] first time reported a novel and simple approach to disperse CNTs completely in silicone rubber by the addition of graphene. Motivated by this, Srivastava and his group made several studies on different combinations of 1D and 2D fillers, together referred as 3D hybrid fillers in fabrication of SR nanocomposites [140,141,142]. First time, they reported fabrication of MWCNT-graphene (G) hybrid (1:1 wt ratio) as nanofiller in enhancing the mechanical properties of high-performance silicone rubber (VMQ) [140]. Table 10 lists mechanical property data of neat VMQ, MWCNT(0.375 wt %)/VMQ, G (0.375 wt %)/VMQ, MWCNT-G (0.75 wt %)/VMQ, MWCNT-G (1.0 wt %)/VMQ, and MWCNT-G (1.5 wt %)/VMQ. These findings showed significant improvements in tensile strength (110%) and Young’s modulus (137%) for MWCNT-G (0.75 wt %)/VMQ composite as compared to neat VMQ. The observed increase in modulus in MWCNT-G/VMQ nanocomposites is in all probability due to the development of shear zones in the nanocomposites under stress and strain conditions, or better dispersion of MWCNT-G leading to its enhanced interaction with VMQ. In addition, EB of VMQ is considerably reduced when filled with 0.375 wt % MWCNTs or graphene. Interestingly, such loss in flexibility of nanocomposites of VMQ individually filled with MWCNTs or graphene is recovered in case of MWCNT-G (0.75 wt %)/VMQ nanocomposite. All of these findings clearly demonstrate the synergistic effect of MWCNT-G hybrid on TS as well as EB of VMQ composites. This could be mainly attributed to the homogeneous dispersion of 3D MWCNT-G hybrid filler in VMQ matrix or due to better interaction between MWCNT-G and VMQ.

Srivastava and his group also extended their work on MWCNT and montmorillonite clay (MMT) in reinforcing properties of silicone rubber nanocomposites [141]. They recorded stress-strain plots for SR, SR/MWCNT (0.5 wt %), SR/MMT (0.5 wt %), and SR/MMT (0.5 wt %)/MWCNT (0.5 wt %). This affirmed that tensile strength in the corresponding composite with respect to SR enhanced by 46%, 25%, and 215% due to the synergistic effect of MMT and MWCNT on SR. They also observed that the Young’s modulus and tensile strength in SR loaded with 1 wt % MMT/MWCNT (1:1) are improved compared to SR by 215% and 133% respectively. The improvements in the tensile strength may be attributed to the homogeneous dispersion of hybrid filler and strong interfacial interaction between nanofillers and SR matrix to transfer the load from polymer matrix to hybrid. The observed increase in Young’s modulus is more likely due to the formation of brittle composite as compared to SR. Alternatively, the possibility of the resistance exerted by the sterically hindered MMT/MWCNT hybrid surface itself and strong polymer filler interaction enhancing the Young’s modulus also cannot be overruled. Elongation at break of SR gradually increases in the presence of MMT/MWCNT (1:1) hybrids and it attains maximum value (260%) at its 1 wt % filler loading. This is in all probability due to the entanglement of the polymer chain/synergistic effect of chain slippage, platelet orientation of MMT, and deformation of the MWCNT. However, TS and EB of SR decrease at higher filler loadings due to the aggregation tendency of the MMT/MWCNT. Motivated by their earlier work, Srivastava and workers [142] also investigated mechanical properties of nanocomposites of SR filled by 3D hybrids consisting of MWCNT-Li-Al-LDH, MWCNT-Mg-Al-LDH, and MWCNT-Co-Al-LDH fillers in SR. It is noted that the tensile strength is maximum improved by 134%, 100%, and 125% as compared to neat SR corresponding to 1 wt % of Mg-Al-LDH/MWCNT, Li-Al-LDH/MWCNT, and Co-Al-LDH/MWCNT, respectively. The role of the synergistic effect of 1D MWCNT and 2D LDH was established based on stress-strain plots neat SR Mg-Al-LDH (0.5 wt %)/SR, MWCNT (0.5 wt %)/SR, and Mg-Al-LDH/MWCNT (1.0 wt %)/SR and similarly for SR composites of other fillers (MWCNT-Li-Al-LDH and MWCNT-Co-Al-LDH). However, the EB of the composites is always less when compared to neat SR. It is also evident that the EB of SR gradually increases in the presence of LDH/MWCNT hybrids and attains maximum value at 1 wt % filler loading. This is in all probability due to the entanglement of polymer chain/synergistic effect of chain slippage, platelet orientation of LDH and deformation of the MWCNT. However, TS and EB of SR decreases at higher filler loadings due to the aggregation tendency of the LDH/MWCNT. In addition to 3 D hybrid fillers, few other combinations of individual fillers have also been used in the reinforcing of SR. SR composite, filled with hybrid fillers consisting of 5 phr CB and 1.0 phr CNT shows improvement in tensile strength and strain to failure due to good dispersion and synergistic effects. Witt et al. [119] prepared SR composites that were filled by MWCNTs, CB, and MWCNT/CB and subsequently studied the mechanical properties of neat SR, SR/3.0CB, SR/4.0CNT, and SR/2.5 CB/1.0 CNT composites. It was noted that SR/ SR/2.5 CB/1.0 CNT composites exhibited have higher tensile strength and strain to failure values than those of SR with single CB or CNT nanofiller of similar concentrations (SR/3.0 CB or SR/4.0 CNT). Norlin and Hazizan [143] prepared PDMS/MWCNT-Al_2_O_3_ (0.5 and 1.5 wt %) nanocomposites by the solvent casting method and examined their mechanical properties. The results showed that lower tensile properties are observed for the greater contents of MWCNT-Al_2_O_3_ hybrid in PDMS.

### 3.5. Mechanical Properties of Carbon Filler Incorporated EPDM and Its Blend Nanocomposites

Ethylene-propylene-dieneterpolymer (EPDM) is one of the most widely used and fastest growing synthetic rubbers because of its excellent resistance to heat, oxidation, ozone, weathering, and microbial attack, which are attributed to the stable and saturated polymer backbone and seals, radiator, electrical insulation, roofing membrane, tubing, belts, and other general-purpose applications, which dominate its impact with regard to various industrial aspects. However, EPDM filled with carbon containing fillers has received relatively lesser attention [144,145,146,147,148,149,150,151,152,153,154,155,156,157]. Enhanced mechanical properties have also been observed in EPDM/ MWCNT rubber composites that are prepared by the solution blending using sonication process [173]. The nanocomposites of EPDM filled with 0.5–5 wt % MWNTs exhibited improved mechanical properties as compared to the pure EPDM matrix [144]. The Young’s modulus significantly enhanced with the increase in concentration of MWNTs. In another study, the tensile strength and elongation at break of compatibilized EPDM/MWCNT (0–7 phr) were found to be higher than those of uncompatibilized nanocomposites. EPDM grafted with maleicanhydride on filling with CNF showed in mechanical strength with little compromise of higher density [147].

Dubey et al. [148] studied the radiation effect on the mechanical properties of SBR/EPDM (50:50) blend containing MWNT (0.5–5%). The elastic modulus, tensile strength increased with the radiation dose, while elongation at break exhibited a downward trend. The extent of reinforcement, as assessed using the Kraus equation, suggested high reinforcement of blend on MWNT addition. They also extended work on effect of radiations on PCR/EPDM/MWCNT nanocomposites [149]. It was noted that mechanical properties are improved due to synergistic effect of MWCNT induced reinforcement and high energy radiation induced crosslinking of the blend. The extent of reinforcement, as assessed using the Kraus equation, suggested high reinforcement of the blend on MWNT addition. The incorporation of silane treated multiwalled carbon nanotubes in EPDM show increase in ultimate tensile strength and hardness [154]. The formation of simultaneously strengthened and toughened nanocomposites based on polypropylene/EPDM matrix was achieved through enhanced adhesion between MWNTs and polymer matrix by using PP grafted MWNTs [155].

The tensile properties of PA-6/EPDM-g-MA (65:35) blend nanocomposites increased with the incorporation of the SWNT [152] The tensile modulus of PP/EPDM was enhanced by increasing SWCNTs contents in EPDM [153]. Narimani et al. [156] studied the effect of SWNT on mechanical properties of thermoplastic elastomer-based polypropylene (PP)/EPDM (80/20). The addition of SWNT increased the storage modulus. The impact strength and tensile strength improved when 0.5% of SWNT was used. Furthermore, the tensile modulus increased remarkably by increasing the SWNT content, but the elongation at break of the material decreased. The mechanical properties of EPDM filled phenol formaldehyde resin coated carbon nanotube have also been studied [157].

Allahbakhsh and Mazinani [201] investigated influences of sodium dodecyl sulfate on the mechanical performance of EPDM/GO nanocomposites. The maximum strength of the nanocomposite was about 137% more in the presence of SDS than the mechanical strength of the EPDM/GO nanocomposite. Furthermore, EPDM/GO nanocomposite was elongated up to 700% in the presence of sodium dodecyl sulfate. Valentini et al. [150] reported tensile properties in terms of the modulus at different strains (50%, 100% and 300%), maximum strength and elongation at break based on stress-strain characteristics of the EPDM- based nanocomposites containing CB, graphene, and graphene platelets (GNPs) [150]. As expected, the addition of the fillers to the EPDM matrix gives rise to an increase of the stiffness of the material, which is reflected in an improvement of the modulus at different strains. The elongation at break decreases on adding GNPs to the EPDM/CB blend. It is also noted that the EPDM/ GNPs (2 wt %)/CB (24 wt %) nanocomposite shows higher increment of the maximum strength along with a higher elongation at break with respect to the EPDM/CB blends due to the synergistic effect of CB and GNPs. Valentini et al. [151] also used platelets GNPs to prepare EPDM nanocomposites and studied its mechanical properties. Their study has shown that the composite with high filler contents give rise to an increase of the stiffness of the material.

### 3.6. Mechanical Properties of EVA Rubber Nanocomposites

EVA copolymers are typically used for electrical insulation, cable jacketing and repair, component encapsulation and water proofing, corrosion protection, and the packaging of components. However, available literature reveals that not much work is reported on the mechanical properties of carbon based nanofiller nanocomposites [158,159]. George and Bhowmick [158] made comparative study of mechanical properties of EVA (40–70% VA content) filled with EG, MWCNT and CNF. They concluded that EVA sample with lowest vinyl acetate content exhibits highest mechanical properties. However, the enhancement in mechanical properties of nanocomposites is the highest for EVA with high VA content. The variation of tensile strength and elongation at break versus EG content (in EVM/Ammonium Polyphosphate/Dipentaerythritol/EG system) decreased gradually with the increase of EG content [159]. Bhuyan et al. [208] observed remarkably improved mechanical properties of neat EVA with HMM content up to 3 wt % followed by reversion. The tensile strength, elongation at break, and toughness showed maximum improvements corresponding to 424%, 109%, and 1122%, respectively. In another study, EVA/MWCNTs/Hectorite (4 wt %) nanocomposites showed significant improvement in tensile strength (243%), elongation at break (105%), and toughness (426%) without significant change in the Young’s modulus.

The literature above clearly signifies god amount of work reported on mechanical properties in MWCNT filled NR, SBR, NBR, SR and their blend nanocomposites compared to EPDM and EVA nanocomposites. In contrast, no such contemporary work is reported on mechanical property improvements in variety of rubbers filled with SWCNT, CNF and graphene oxide. Further, it would be more interesting to focus on the evaluation of mechanical properties in rubber composites consisting of 3D hybrid nanomaterials due to the synergistic of the individual nanofillers 

## 4. Dynamical Mechanical Properties of Carbon Nanofillers Containing Rubber Nanocomposites

The storage modulus reflects the elastic modulus of the rubber materials, which measures the recoverable strain energy in a deformed specimen, and the loss factor is related to the energy damped due to energy dissipation as heat. Therefore, the dynamic mechanical property of different rubbers containing carbon nanofillers has been studied. The storage modulus, loss modulus, and glass transition temperature increased for all MWCNTs reinforced NR [24]. In another work, the addition of CNT at a very low loading could enhance the storage modulus of NR/SBR and NR/XSBR blend nanocomposites prepared by latex compounding [61].

Boonmahitthisud and Chuayjuljit [61] studied variation of storage modulus and loss tan *δ* as a function of temperature for neat NR/SBR and NR/XSBR blends and nanocomposites filled with varying amount of CNT. These findings indicated that dose-dependent increase in the E′ in CNT added rubber blend nanocomposites as compared to the neat rubber blends and ascribed this to the high stiffness of CNT that constrains the rubber chain motion. Further, the T_g_ of each rubber in NR/SBR and NR/XSBR nanocomposites increased with the increase in CNT loadings. Thomas et al. [62] showed shifting of T_g_ to a higher temperature in the case of NR/phenol functionalized CNT nanocomposites. Loss tangent showed a decrease in the presence of CNT, and the effect is more pronounced in the case of phenol functionalized CNT. Sui et al. [47] observed that the storage modulus of the CNT/NR nanocomposites greatly exceeds that of neat NR and CB/NR composites. The glass transition temperature of natural rubber/ethylene propylene diene monomer nanocomposites slowly enhanced with increasing the contents of MWCNT [56]. Xu et al. [32] performed dynamic mechanical measurements on NR/CB composite and NR/CB/MWCNTs composites with different MWCNTs loadings. Thermoplastic PP-NR blend filled with acid-treated MWCNTs has the highest storage modulus (−90 °C), while the TPNR containing untreated MWCNTs has the lowest [61]. This could be in all probability due to the fine dispersion of the acid treated MWCNTs in the TPNR matrix. Bhattacharya et al. [65] observed superior dynamic mechanical properties of NR/CNF nanocomposites when compared with those to either NR/clay or NR/CNF nanocomposites or the NR/black control microcomposite. Natural rubber/graphene nanocomposites that were prepared by direct mechanical mixing showed improved dynamic properties [48]. The elastic modulus of NR at room temperature increases and the maximum loss tangent and the corresponding glass transition temperature of composites decrease with increasing content of MWCNT [160,161]. The storage modulus of the NR/graphene oxide and nanocomposites significantly increased with the GO contents, indicating that GO had a strong reinforcing tendency on NR [202]. When compared with neat NR, modulus at 300% for NR composites containing 0.9 phr graphene was increased by 154.9% [187]. The improvements in dynamic mech. properties were achieved at small substitution content of GO or reduced grapheme nanosheets for carbon black in NR.

The rolling resistance and wet traction properties of SBR are very valuable for their applications, especially in tire trade. In view of this, the dynamical mechanical analysis of SBR filled with carbon-based nanomaterials received attention [15]. Peddini et al. [68] measured storage modulus, loss modulus, and tan δ versus temperature (−60 to 60 °C) for the original masterbatch (12.3 wt % MWCNT-SBR) and various dilutions with SBR. They noted that storage modulus and loss modulus increase with the addition of MWCNT in the composite. T_g_ of the cured plaques by DMTA also increase 4.5 °C with the dilution of the SBR masterbatch containing 12.3 wt % MWCNT. In another work, Pedroni et al. [70] compared the DMA findings of SBR/MWCNTs nanocomposites made by the solution casting and melt mixing methods. Storage modulus increases with the amount of filler in SBR in either method due to its reinforcing effect, though effect being more evident for composites that were prepared by the casting method. The T_g_ of the polybutadiene blocks (PB) in the composites prepared by the casting method shifts from −88 to −80 °C with increasing MWCNT content. In contrast, no significant change in the T_g_ was observed for PB blocks of the composites prepared by extrusion. The addition of CNTs in SBR/BR (50:50) blend affects the glass transition behavior [73]. Adohi et al. [82] observed that the storage modulus of the SBR filled with carbon black- and carbon nanotubes composites is close to the modulus value of the neat styrene-butadiene rubber (f < 0.1). Dynamic mechanical properties of modified expanded graphite/emulsion styrene butadiene rubber nanocomposites demonstrated an improvement when compared to the respective control [86] Zhang et al. [87] reported dynamical mechanical properties of SBR/CB and SBR/CB-RG hybrid filled nanocomposites, T_g_ is shifted slightly to higher temperature on adding CB from 10 phr to 13 phr in SBR. In contrast, T_g_ shifted to a higher temperature by increasing the RG loading in RG-CB hybrid filled SBR blend nanocomposites. The comparison of two blends with the same total filler loading in SBR suggested that SBR/CB-RG (100/10:3) exhibited higher T_g_ than that of SBR/CB (100/13). Tang and coworkers [89] noted that tubular clay (HNT)-tannic acid functionalized graphene (TAG) hybrid (22 phr) showed relatively higher modulus with respect to individually filled 20 and 2 phr of HNT and TAG, respectively. The corresponding value of T_g_ were found to be −34.3, −37.9, and −32.2 °C, respectively. Dynamical mechanical analysis indicated that the storage modulus of the composites was improved with the CNTs addition, especially CNTs. exceeding 30 phr in SBR [163,164]. Liquid polyisoprene and MgO affected the dynamical mechanical properties of SBR/CNT vulcanizate, increasing the elastic modulus, glass transition temperature, and tan δ value at 0 °C [165]. Another study showed a reduction in T_g_ and enhanced the storage modulus of SBR matrix in the presence of PVP-modified GO [206]. Recently, Bhuyan et al. [207] observed no significant enhancement in the dynamic mechanical properties of SBR/MWCNT/Hectorite nanocomposites.

The significant increase in storage modulus of NBR was observed in the presence of relatively much lower volume fraction of CNTs when compared to carbon black [95]. Likozar and Major [100] reported that storage modulus (25 °C) is enhanced by 6.0–8.3 times in NBR/MWCNT compared to HNBR/MWCNT, but the enhancement for 34.0 wt % AN content nanocomposite is only 3.3–4.8 times. Mahmood et al. [110] studied the effect of CNTs loading on the loss tangent (tan δ) of TPUU:XNBR blend system as a function of temperature.(−60 to 100 °C,10 Hz). It is noted that the intensity of the tan peak decreases with increasing the amount of the CNTs in the TPUU/XNBR. These findings also show no significant shift of the tan d peak at about 25.8 °C on adding of the CNTs. The observed changes in the shape and intensity of the tan peaks in TPUU:XNBR/CNT blend nanocomposites in all probability due to extent of the interaction/distribution of the CNTs. Salehi et al. [107] observed improvements in the dynamic storage modulus, E′ (25 °C) and a decrease of the magnitude of the loss angle (tan δ), of SBR/CNT/SiO_2_ 25/3 phr (E′ = 8.2 MPa, T_g_ = −1 °C) and NBR/CNT/SiO_2_ (25/5 phr) (E′ = 9.6 MPa, T_g_ = 0 °C) as compared to neat NBR (E′ = 1.6 MPa, T_g_ = −1.4 °C) due to good polymer-filler interaction including synergistic effect. PVC/NBR filled with cylindrical SWNT exhibited improved storage modulus [110]. Srivastava and his group [112] observed significant improvements in the dynamical mechanical properties of NBR/TPU blend in presence of Zn-LDH/surfactant modified CNT (referred as (SFCNT) and surfactant modified CNF hybrid filler. The storage modulus and loss modulus of hybrid filled NBR/TPU nanocomposites were found to be always higher compared to neat blend and blend filled with 0.50 wt % hybrid loading exhibited maximum improvements. They also extended their dynamical thermal analysis on SFCNF/Mg-Al-LDH filled neat NBR/TPU nanocomposites [113]. It is noted that 0.50 wt % SFCNF-LDH filled NBR/TPU achieved maximum E’ (276% at −60 °C, 261% at 25 °C) and loss modulus (254%, at −30 °C), including maximum positive shift in T_g_ (~3 °C) as compared to the neat sample. Dynamical mechanical properties of TPU/NBR/SFCNT/Mg-Al-LDH blend nanocomposites were also carried out in the range −80 to 80 °C [114]. The storage modulus of the 0.50 wt % hybrid filled TPU/NBR matrix is significantly increased in both the glassy region (by 243% at −60 °C) and rubbery state (by 241% at 25 °C) compared to pure TPU/NBR. Such enhancement in the storage modulus is attributed to the homogeneous dispersion of the SFCNT-LDH nanofiller within the matrix along with strong interaction between SFCNT-LDH hybrid filler and polymer matrix, which accounts for stress transfer from the TPU/NBR matrix to the hybrid filler, resulting in strong reinforcement. The loss modulus (at −30 °C) improved by 254% for TPU/NBR nanocomposites filled with 0.50 wt % hybrid, as compared to pure TPU/NBR.

The dynamic storage modulus of NBR/EG composites is found to be significantly higher than that of pure NBR rubber about one order of magnitude above the glass transition temperature [101]. This is ascribed to the good reinforcing effect of nano-size graphite and the restricted chain mobility of the NBR chain segments. Liu et al. [102] observed a higher storage modulus and lower glass transition temperature in NBR/EG NBR/EG composites. The storage modulus and loss factor (tan δ) of the NBR/EG/CB nanocomposite were relatively higher than those of NBR/EG and NBR/CB [103].

**Figure 19:**
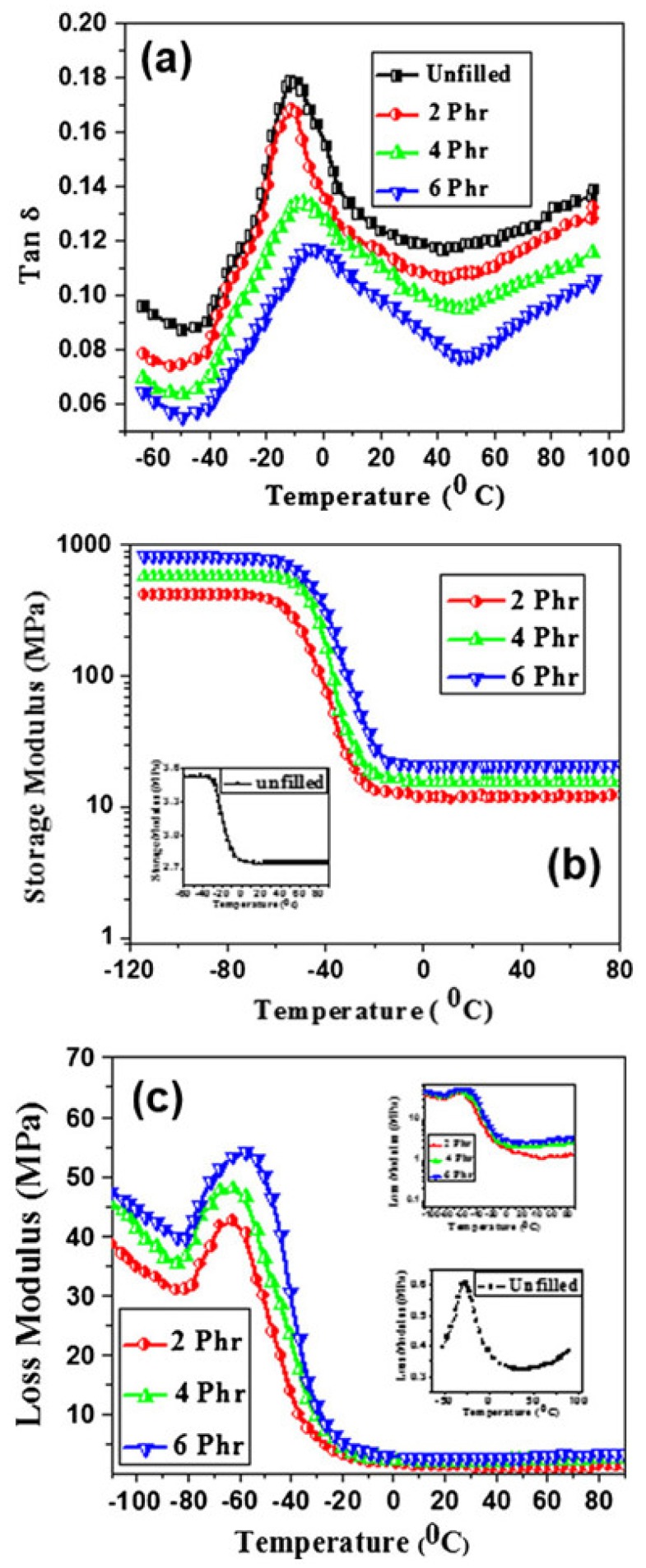
(**a**) Variation of loss tangent (Tan δ) with temperature as a function of MWCNT loading in silicone elastomer nanocomposites; (**b**)Variation of storage modulus with temperature as a function of MWCNT loading in silicone elastomer nanocomposites; (**c**) Variation of loss modulus with temperature as a function of MWCNT loading in silicone elastomer nanocomposites. Reproduced with permission from [129], Copyright Springer, 2014.

PDMS/silane molecules onto diphenyl-carbinol-functionalized MWCNT nanocomposites showed enhanced dynamic mechanical properties when compared to those containing unmodified MWNTs and diphenyl-carbinol-functionalized MWNTs [115]. Saji et al. [129] studied effect of MWCNT loadings on loss tangent, storage modulus, and loss modulus, and the corresponding findings are shown in Figure 19. It is noted that the location of maximum value of loss tangent (tanδ_max_) is not significantly affected by the extent of MWCNT loading. The glass transition of all the samples lies in the range of −10 °C to −5 °C. This can be explained on the basis of relaxation dynamics of the polymer matrix. The variation in storage modulus (E′) as a function of temperature shows characteristics of sigmoidal variation. The temperature dependence of the loss modulus (E″) showed the appearance of a distinct peak (α transition) at ~−50 °C for all filled compositions. This could be ascribed to conformational transitions occurring in the silicone elastomer backbone caused by micro-Brownian motion. Roy and Bhowmick observed no prominent shift in the glass transition temperature shift of PDMS (−116 °C) and its amine modified CNF filled PDMS nanocomposites. The effects of the incorporation of SWNTs on the dynamical mechanical properties of blends of isotactic polypropylene (iPP) and EPDM are investigated [166]. The effect of the incorporation of SWNT increased the storage modulus of PP/EPDM (80/20) thermoplastic blend [156].

## 5. Payne and Mullin Effects in Carbon Nano Fillers Incorporated Rubber Nanocomposite

The Payne and Mullins effect describe the changes in the properties of an elastomer component as a function of the applied strain [223]. The Payne effect originates from the stress-strain behavior of rubber, especially rubber containing fillers, such as carbon black and named after British rubber scientist A. R. Payne [168,224]. The amplitude variations in the storage and loss modulus, the so-called Payne effect, are general to all filled elastomers [225]. The Payne effect manifests as a dependence of the viscoelastic storage modulus on the amplitude of the applied strain [226]. This effect could be attributed to deformation-induced changes in the microstructure of the material [227]. Similar to the Payne effect under small deformations is the Mullins effect that is observed under large deformations. This effect remains a major challenge in order to provide good mechanical modeling of the complex behavior of industrial rubber materials [228]. The Mullins effect is closely related to the structural changes during the tensile cycles. The most commonly accepted model to explain Payne effect arises from the breakdown of a filler network formed by filler–filler interactions.

**Figure 20:**
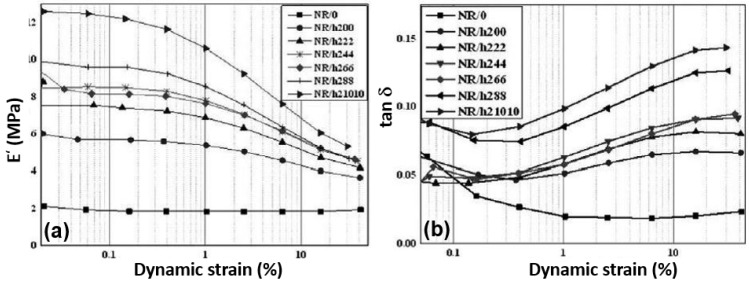
Strain dependence of E′ (**a**) and tan δ (**b**) for the NR/0 and NR/MWCNT/EOMt nanocomposites. Reproduced with permission from [44], Copyright Elsevier, 2015.

**Figure 21:**
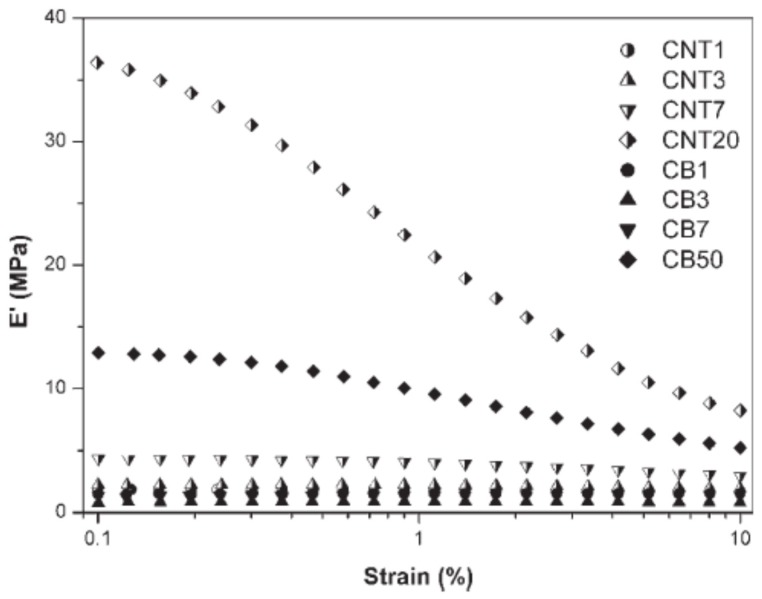
Storage modulus of NR/CNT and NR/CB compounds at different filler loadings as a function of dynamic strain amplitude. Reproduced with permission from [170], Copyright Wiley, 2010.

**Figure 22:**
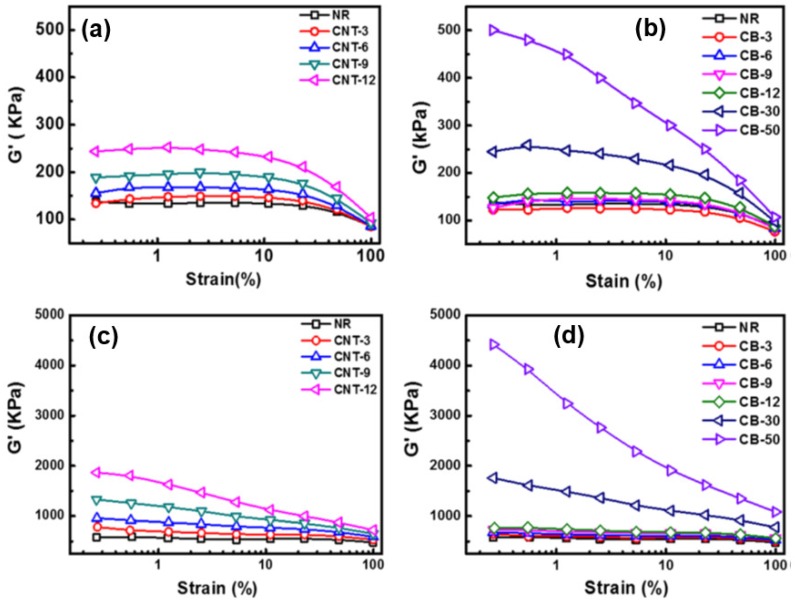
The strain dependence of G′ of CNT/NR and CB/NR rubber compounds (**a**,**b**) and vulcanizates (**c**,**d**). Reproduced with permission from [172], Copyright Springer, 2017.

Dong et al. [35] studied variation of storage modulus as a function of dynamic strain amplitudes at 0, 1, 3, 5 phr loadings of carbon nanotube bundles (CNTB) in NR. It is noted that CNTB (5 phr) filled NR shows the highest storage modulus and magnitude of the Payne effect. Galimberti et al. [36] observed that the Payne effect increased with the CB-CNT and G-CNT nanofiller contents in synthetic poly(1,4-cis-isoprene). Ivanoska-Dacikj et al. [44] studied the dynamic mechanical properties of NR nanocomposites containing 2 phr MWCNT and different quantities (from 0 to 20 phr) of expanded organically modified montmorillonite (EOMt). Figure 20 show strain sweep measurements by applying cyclic deformations in the tension mode to determine E′ (storage) and E″ (loss) moduli respectively. The dependence of E′, as measured at low strain (so called Payne effect), on the hybrid filler content was studied to investigate the existence of a percolation threshold in the NR matrix. It showed a significant increase of the loss factor tan δ for the nanocomposites with filler concentrations above the mechanical percolation threshold (16 phr). This percolation threshold was found to be much higher when compared to the NR/EOMt system without MWCNT. This study also indicated pronounced non-linear dependence, called the Payne effect, and could be explained in terms of a filler–filler network in the polymer matrix above the filler percolation threshold. Tan δ versus temperature plots in Figure 20 also indicated a remarkable increase of the loss factor tan δ for the nanocomposites NR/MWNT (2 phr)/EDMt (16 phr) and NR/MWNT (2 phr)/EOMt (20 phr) at higher dynamic strains. This effect could be attributed to the filler–filler network breaking down. Nah et al. [170] investigated the variation of storage modulus of NR filled with varying loadings of CNT and CB (1, 3, 7, 20 phr) as a function of dynamic strain amplitude and the findings are displayed in Figure 21. It is noted that storage modulus of filled rubber compounds decreases significantly in contrast to little changes displayed in unfilled rubbers. This nonlinear behavior at small strains in NR/CNT nanocomposites is known as the Payne effect. It is more pronounced at higher filler loadings and is significantly higher in the CNT-filled compounds. Figure 21 also show hysteresis loops of NR/CNT and NR/CB compounds with 1 phr of filler content. This clearly demonstrates tress softening phenomenon at large strains (Mullins effect). It is concluded that Payne effect and Mullins effects are more dominant in CNT filled NR than CB-filled NR. Strain dependence of the storage modulus of NR and NR–MWCNTR has also been reported by George et al. [171], where Payne effect was not observable below 0.3 phr MWCNTR. Above that, the effect increases with increase in the amount of MWCNTR in NR. Yang et al. [172] studied strain dependence of storage modulus (shear mode) of carboxylated CNT/NR and CB/NR rubber compounds and vulcanizates, as displayed in Figure 22. It is inferred that the storage modulus decreases with strain increasing in CNT/NR as well as CB/NR due to the Payne effect. This effect is also more inevitable with an increasing in SR. They observed constant storage modulus corresponding to 3 phr of CNT or CB 3–9 phr of CB in NR. However, the storage modulus increased remarkably for CNT content of 9, 12 phr and CB content of 30, 50 phr in CNT/NR and CB/NR, respectively.

Kang et al. [173] carried out strain sweep measurements of graphene/NR nanocomposites that were prepared by direct mechanical mixing. The variation of storage of these filled compounds shows non-linear behavior and high sensitivity to strain (Payne effect). Further, it is noted that the storage modulus greatly increased with the addition of graphene due to the formation of filler network. Aziz et al. [175] investigated variation of storage modulus versus strain amplitude of MRE filled with different types of MWCNT at different magnetic fields (0, 156, 331, 508, 638, and 747 mT). These findings showed that storage modulus gradually dropped as the strain amplitude increases. Such non-linear behavior of filled rubber, i.e., Payne effect, can be explained in terms of filler network in the rubber matrix above the percolation threshold.

**Figure 23:**
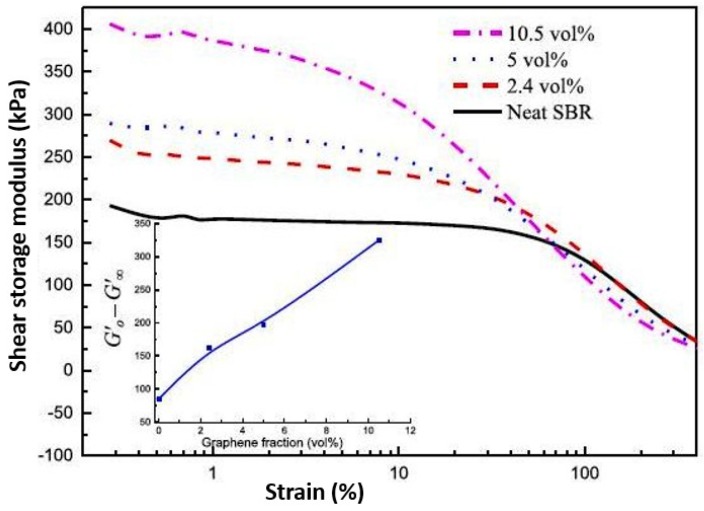
Payne effect of elastomer and its nanocomposites. Reproduced with permission from [174], Copyright Elsevier, 2014.

Araby et al. [174] studied variation of shear storage modulus versus strain (%) of SBR filled GNP nanocomposites and findings are displayed in Figure 23. It showed a decrease of shear storage modulus with an increase in the applied strain amplitude. The inset of the Figure 23 clearly indicates the overall proportional increase of the Payne effect with GNP fractions. This is ascribed to the formation of filler-filler network in an elastomer matrix. In another study, storage modulus of SBR-HG compounds decreases dramatically upon increasing strain (Payne effect) [89].

Katihabwathe et al. [125] studied the dynamical mechanical behavior of uncured CNT/SR (1, 5, 10, and 20 wt %) nanocomposites. They observed that storage modulus decreases in the filled rubbers with increasing strain amplitude, i.e., characteristic of the phenomenon, which is known as the Payne effect. Further, this effect decreases with decreasing amount of filler and is not observed for CNT < 5 wt %. However, Payne effect is pronounced for SR filled with 20 wt % or more, as evidenced by significant changes in storage modulus with increasing strain amplitude. Li and Sun [130] studied effect of pre-strain levels (2%, 5%, and 8%) on the dynamic mechanical properties of MWCNT-reinforced silicone rubber with 5 wt % MWCNT loading in a dynamic strain sweep from 0.1% to 10% at frequency of 10 Hz. This study showed that the loss modulus and tan delta are not clearly dependent on the pre-strain level. However, higher pre-strain level leads to slightly higher storage modulus over the entire dynamic strain sweep for each nanocomposite.

The decrease in storage modulus of nanocomposites is noted with increasing dynamic strain amplitude, while silicone rubber showed very small change. This effect is more pronounced at high MWCNT loading exhibiting a non-linear behavior. The dependence on dynamic strain amplitude of dynamic mechanical properties of SR filled with CNT (1, 2, 3.5 and 5 wt %) at 10 Hz showed decrease in storage modulus of nanocomposites with increasing dynamic strain amplitude [130]. This effect is more pronounced at high MWCNT loading, showing a non-linear behavior (Payne effect). Hoikkanen et al. [111] observed that MWCNT filled EPDM/NBR blend nanocomposites that were prepared by the direct mixing method showed higher Payne effects than those prepared by the masterbatch dilution method.

Room temperature variation of storage modulus versus strain amplitude (%) of the NR filled with 1 and 5.4 wt % carboxylated MWCNT [169]. It is noted that the storage modulus of NR nanocomposite decreases when the strain amplitude increases. Loading-unloading cycles in cyclic tensile tests showed a strong stress-softening (Mullins) effect, especially at 8.3 wt % CNT filling. The observed strong Payne effect and strong Mullins effect are ascribed to the mechanical behavior of the nanotube network.

NR and SBR find wide applications in automotive tires components, conveyer belts, electrical wires, cables, and many other industrial products. However, essential requirements for satisfactory performance of these products demand good mechanical properties. In view of this, more work is needed to be focused on the dynamical mechanical properties of NR and SBR and its blend nanocomposites filled with modified CNT, CNF, graphene, and hybrid nanofillers. Similarly, NBR, SR, EPDM, and EVA also have plenty of scope in investigating the dynamical mechanical properties of their carbon filled rubber nanocomposites.

## 6. Summary and Outlook

This review article has addressed recent research carried out on elastomers that are filled with carbon nanomaterials. The melt mixing and solution methods are the most widely used methods to prepare polymer nanocomposites. The state of dispersion of fillers accounts for the enhanced mechanical and dynamical mechanical performances of individual carbon based fillers, such as CNT, CNF, graphene, expanded graphite, and graphene oxide. In addition, improvement is also pronounced with the hybrid fillers consisting MWCNT (or CNF)-MMT, MWCNT-Hectorite, MWCNT (or CNF)-LDH, and MWCNT-Graphene in a variety of rubbers matrix. It is inferred that the introduction of these carbon based fillers in NR, SBR, NBR, and SR nanocomposites led to significant improvements in tensile strength, impact strength, elongation at break, Young’s modulus, stand loss modulus, storage modulus, etc. However, more contemporary work is still to be focused on the development of corresponding EPDM and EVA nanocomposites. Payne and Mullin effects in rubber- carbon fillers nanocomposites are also reviewed. Thus, unique properties of carbon-based fillers make them superior when compared to conventional fillers, such as carbon blacks and silica requiring higher filler loading to enhance the mechanical properties of polymers. However, clear inference is not possible to compare the mechanical and dynamical properties of different nanostructural carbon filled individual rubbers due to their dependency on state of filler dispersion.

## Figures and Tables

**Table 1:** Types of carbonaceous nanofillers, preparative methods adopted and morphology of natural rubber (NR) nanocomposites.

Rubber	Filler	Preparation Method	Morphology	Ref.
NR	MWCNT	Solution method	SEM: Contact between Fe_3_O_4_ and MWNTs increases with the increasing filling fraction of particle.	[21]
TPNR	MWCNT	Melt blending	SEM: 3 wt % of MWCNT is well dispersed in TPNR matrix	[22]
NR	MWCNT	Master batch mixing method (open two roll mill)	SEM: CNT bundles totally exfoliated in CNT (1 phr) filled NR, uniform CNT (3 phr) dispersion and agglomeration of CNT (5 phr) bundles.	[23]
TPNR	MWCNT	Melt blending	SEM: Good dispersion of MWNTs in TPNR after sonication for 1 h.	[24]
NR	CNT	Mixing method	TEM: Large agglomerates in microscopic level in CNT (5 phr) filled NR (without ethanol).	[25]
NR	Functionalized CNT	Melt blending	—	[26]
L (Liquid) NR	MWCNT	Melt blending	SEM: Good dispersion of MWNTs (3.5 wt %) in PLA/LNR; Poor dispersion and agglomeration of MWCNTs (4%) in PLA/LNR.	[27]
NR	CNT	Solvent casting method	TEM: Good dispersion of CNT (3 phr) in SBR/BR (1:10 ethanol).	[28]
NR	MWCNT	Solvent casting method	TEM: 1 wt % of CNTs are homogenously distributed in the NR matrix	[29]
NR	SWNTs	Latex stage mixing method	—	[30]
NR	MWCNT	Ultrasonically aided extrusion	AFM: Deagglomeration of MWCNT (3.5 phr) at an amplitude of 7.5 μm.	[31]
NR	CB-MWCNT	Latex compounding	SEM: MWNTs uniformly dispersed in NR matrix	[32]
NR	Silica/MWCNT	Two-roll mill	SEM: Good dispersion and better interaction between silica and the MWCNTs with the NR matrix at a 29/1 silica/MWCNT loading ratio	[34]
NR	CNT/CB	Mechanical compounding method	TEM: Homogeneous dispersion of CNT in CNTB (1 phr)/CB (22 phr), CNTB (3 phr)/CB (16 phr) and CNTB (5 phr)/CB (10 phr) and formation of hybrid filler network.	[35]
NR	CNT, Graphene, CB-CNT	Melt blending	—	[36]
TPNR	MWNTs-OMMT	Melt blending	TEM: Good dispersion of OMMT-MWNTs (3 h) in TPNR	[37]
TPNR	MWNTs-OMMT	—	TEM: Indicated strongly to interfacial adhesion between fillers and the matrix	[38]
NR	Graphene /CNT	Modified latex mixing method	SEM and TEM: Homogeneous dispersion of graphene (5 phr) and CNT (5 phr) in NR matrix.	[39]
ENR	CNT	Mixing in internal mixer, roll milling	SEM: ENR-CNT composites with APTES additions and 0.01 mL/(g of CNTs) at 5 phr CNT loading.	[40]
ENR	Bis(triethoxysilylpropyl) tetrasulfide (TESPT)functionalized MWCNT	Melt blending	SEM: ENR-CNT and the ENR-CNT-TESPT composites confirmed good dispersion of the CNTs in the ENR matrix.	[41]
ENR	CNT	Latex technology	TEM: Good dispersion of CVNT in ENR matrix.	[42]
NR	VGCF	Solvent casting	SEM: VGCFs (1, 3 and 10 wt %) dispersed randomly and evenly in NR matrix.	[43]
NR	MWCNT/organically modified montmorillonite	Internal mixing	TEM: Dispersion of the MWCNT throughout the NR matrix in NR/MWCNT (2 phr); Agglomerates of both MWCNT and EOMt and regions with fully exfoliated structure in NR/MWCNT (2 phr)/Modified montmorillonite (20 phr)	[44]
NR latex	MWCNT treated with SDS	Combination of the latex compounding and self-assembly	MWCNT–PDDA prepared with the self-assembly technique was more intimate with the NR matrix	[45]
NR	CNT	Solution casting	SEM: CNTs well distributed into NR	[46]
NR	CNT	Solvent mixing assisted with a two-roll mill	SEM: Dispersion of the CNTs in rubber matrix.	[47]
NR	MWCNT, Graphene nanoplatelets	Roll milling	—	[48]
NR	Functionalized graphene oxide	Two roll mill	Good dispersion of FGS jn NR matrix	[49]
ENR	MWCNT	Mixing in an internal mixer,	TEM: More homogeneous dispersion and distribution of S-MWCNTs in the ENR matrix.	[50]
NR	MWCNT	Latex compounding	—	[51]
NR	MWCNT	Solvent mixing method	TEM: Homogeneous distribution of MWNTs (3 phr) in NR matrix.	[52]
ENR	MWCNT	Roll mill	SEM: Distribution of MWNT (4 phr) are observed in ENR matrix.	[53]
NR	CB/MWCNT	Mixing in laboratory two roll mill	SEM: A good dispersion is seen corresponding to CB/MWCNT (29.5/0.5) loading.	[54]
NR/XSBR	CNT (0.1–0.4 phr)	Latex compounding	—	[58]
TPPU	(MWCNTs)-magnetite (Fe_3_O_4_) hybrid	Internal mixer using melt blending method with ball-milling as a pre-mixed process.	—	[60]
NR/SBR NR/XSBR	CNT	Latex compounding method	—	[61]
NR	CNT	Solvent casting method	TEM: Phenol functionalization causes improvement in dispersion of CNT in NR matrix	[62]
NR	MWCNT	Solution blending	TEM: Good levels of dispersion with well-isolated nanotubes in NR/MWNTs (4 phr) composite.	[63]
NR	Carboxylated MWCNT dispersed with SDS	Mixing, sonicating and casting	AFM: No aggregates of MWCNT (2.8 wt %) observed in NR.	[169]
NR	MWCNT	Internal mixer, roll mill	SEM: CNTs well-dispersed (unstretched state) in NR/MWCNT (7 phr).	[170]
NR	MWCNT	Ultrasonication assisted latex mixing and film casting method	TEM: MWCNTs wrapped around NR particles forming a segregated network.	[171]
NR	CNT	Mechanical blending	SEM: CNT (9 phr) are disperses well in NR matrix.	[172]
NR	Graphene	Direct mechanical mixing	SEM: Graphene (2 phr) well dispersed in NR.	[173]
NR	Modified NWCNT	Sonication, mixing roll milling	SEM: COOH-MWCNT and CIP have better compatibility among MRE samples. This leads to the formation of interconnected network in the matrix	[175]
NR	RGOT (Thermally reduced graphene oxide)	Mixing/two roll milling	TEM: NR vulcanizates filled with RGOT at 5 phr exhibited networks of aggregated or agglomerated filler networks with a decreased gap between the filler aggregates or agglomerates.	[177]
NR	Polyvinyl pyrrolidone modified graphene oxide PGO)	PGO aqueous dispersion with NR latex, followed by coagulation and vulcanization	SEM: The fracture surface of GO5 has more irregular tear paths suggesting the greater tear strength while PGO5 more tears paths with irregular branches.	[178]
NR	Graphene oxide	Latex mixing/two-roll milling	TEM: Uniform dispersion of graphene oxide (1 phr) in NR/GO	[179]
NR	Graphene oxide nanosheets (GON)	Solution blending	SEM: GON (5 wt %) homogeneously dispersed in NR matrix.	[180]
NR	Graphene oxide	Latex mixing	—	[181]
NR	Thermally reduced graphite oxide (TRGO)	Mixing method	TEM: TRGO prepared by the Brodie’s method (TRGO-B) showed more homogeneous dispersion and distribution through the NR matrix.	[182]
NR	Thermally reduced graphite oxide (TRGO)	Latex method	TEM: Graphene sheets in TRGO dispersed in SDS are distributed throughout the NR.	[183]
NR/HDPE	Graphene oxide	Ultrasonically assisted latex mixing process	SEM: Delamination of the GO sheets in NR/HDPE is observed. Mainly GO sheets are dispersed in the NR phase.	[184]
Grafted NR (GMA)	Graphene Oxide	Mechanical mixing method.	SEM: GO (3 and 6 phr) dispersed uniformly in the NR-g-GMA.	[185]
NR	Functionalized graphene oxide grafted by maleicanhydride grafted liquid polybutadiene (MLPB-GO).	Co-coagulation process.	FESEM: GO sheets dispersed as single layers in NR/MLPB-GO (2.12 phr)	[186]
NR latex	Reduced graphene oxide (rGO)	Ultrasonically assisted latex mixing and the co-coagulation	HRTEM: rGO (0.9 phr) presents a good dispersion and exfoliation in NR.	[187]
NR	ZnO nanoparticles doped graphene	Two roll mill	FESEM: Good dispersion of graphene in the matrix and strong adhesion between graphene and NR in nanocomposite.	[188]

**Table 2:** Types of carbonaceous nanofillers, preparative methods adopted and morphology of SBR nanocomposites.

Rubber	Filler	Preparation	Morphology	Ref.
SBR (25% styrene Content)	Ionic liquid modified MWCNT	Two roll mixing mill.	TEM: A substantial dispersion of modified MWCNT with less agglomerates in SBR	[64]
Solution-SBR/BR	MWCNTT	Roll mill	TEM: Smaller magnification show no homogeneous distribution of the MWCNTs (5 phr)	[65]
SBR (23.5 wt % bound Styrene)	MWCNT	Solvent casting method	FE-SEM: MWCNT (0.66 wt %) are dispersed homogeneously in SBR.	[66]
SBR	MWCNT	Solvent casting	-	[67]
SBR	MWCNT	Coagulation method followed by mastication in a twin-screw extruder	SEM: MWCNT (15 wt %) are well dispersed in the masterbatch.	[68]
SBR	MWCNT	Coagulation process followed by melt mixing	—	[69]
SBR	Functionalized CNT	Solvent casting	—	[71]
SBR and butadiene blend	Modified/unmodified MWCNT	Solvent casting	TEM: CNT form percolating networks at 5 phr loading.	[73]
SBR	Functionalized CNT	Solvent casting	—	[74]
SBR (25% styrene content)	MWCNT	Two roll mixing method	TEM: Good dispersion of MWCNT in SBR matrix	[75]
SBR	MWCNT	Spray drying/mechanical mixing process.	SEM: Dispersion of MWCNTs (50 phr) in the rubber matrix remarkably improved.	[76]
SBR (23% styrene and 77% butadiene)	CB, CNT, Carbon Graphene, Graphite	In banbury mixer	—	[77]
SBR Latex (20 wt % styrene, 80 wt % butadiene)	Graphene(MLG350), CRGO, and TRGO	Latex blending techniques	TEM: SBR nanocomposites containing 25 phr MLG350, CRGO, and TRGO homogeneously dispersed in the SBR.	[79]
SBR (styrene content of 23.5%)	CNF	Mixing followed by two-roll milling	TEM: Fiber diameter (unmodified) of 78 nm swells to 97 nm in SF6 (unmodified SBR) and 144 nm in TSF6 (modified SBR)	[81]
SBR(23.5 wt % styrene SBR polymer)	CNT	Mechanical mixing	TEM: Fairly good dispersion.	[82]
SBR Latex (21 wt % of SBR content)	Graphene	Modified latex compounding method	TEM: Exfoliated graphene (7 phr) is homogeneously dispersed in SBR.	[83]
SBR latex (21 wt % of SBR content)	Graphene	Mechanical stirring	—	[84]
XSBR (23% styrene content)	Modified expanded graphite	Solution mixing and melt blending	HR-TEM: MEG nanosheets uniformly dispersed in the XSBR matrix.	[86]
SBR	CB-Graphene	Two roll mixing	Homogeneous dispersion of graphene sheets on the application of hybrid CB-RG filler in SBR matrix.	[87]
SBR	MWCNT/Thermally reduced graphene (TrG)	Two roll mill	SEM of tensile fracture surfaces: Excellent dispersion of CNTs (1 phr)/TrG (0.25 phr) in SBR matrix.	[88]
SBR latex	Tubular clay and (HNT) functionalized graphene (TAG)	Dispersion of HNT and HNT-TAG hybrid filler added into SBR latex.	TEM: HNTs (40 phr) individually dispersed and as aggregates in SBR TAG (4 phr) uniformly dispersed in SBR. Dispersion of HNTs is greatly improved in the presence of TAG (HNT-TAG:44 phr) in SBR.	[89]
SBR	Fe-MMT/MWCNT	Mixing method	TEM: MWCNT/MMT (5 phr) filled SBR composite show CTAB-MWCNT networks are disrupted by the well dispersed FE-MMT particles.	[90]
SBR	CNT	Spray drying of the suspension of CNTs in SBR latex.	SEM:SBR/CNTs (20 and 50 phr) powder is uniformly spherical (Diameters < 10 μm). On adding more CNTs, powders are more spherical-like and the isolation among the spherules is improved as well.	[163]
SBR	Graphene platelets(GNPs)	Solution mixing, melt compounding	TEM: Better dispersion and exfoliation of GNP (2.4 vol %) in the matrix in solution-prepared nanocomposite, while GNPs exist as aggregates of stacked in the melt-prepared samples.	[174]
SBR	Ionic liquid functionalized graphene oxide (GO-IL)	Latex hetero-coagulation method	SEM: GO-IL can be well-dispersed in the SBR matrix	[191]
SBR latex	3D segregated graphene	Mixing	TEM: 3D segregated graphene networks throughout SBR matrix.	[192]
SBR	MWCNT/Hectorite	Solution blending	FESEM (fracture surface): homogenous dispersion in 0.70 wt % MWCNT/ Hectorite.	[207]

**Table 3:** Types of carbonaceous nano fillers, preparative methods adopted and morphology of NR nanocomposites.

Rubber	Filler	Method of Preparation	Morphology	Ref.
NBR/PA6	Functionalized SWCNT	Melt mixing process in internal mixer	TEM: homogenous dispersion of SWNT in the PA6 phase	[91]
NBR/PVC blends	SWCNT	Brabender internal mixer	TEM: Fine dispersion of functionalized SWNTs (1.5 phr) is observed NBR/PVC.	[92]
NBR	CNT	Lab mixing	—	[93]
HNBR	CNT	Roll and mixing solvent methods	TEM: The modified CNTs disperse very well in HNBR when the ultrasonic pre-dispersing technique is utilized to prepare composites.	[94]
NBR	MWCNT	Two roll mill	TEM: Dry mixing process is quite effective to disperse and distribute CNTs in NBR.	[95]
NBR	MWCNT	Two roll mill	TEM: MWCNT (6 phr)/NBR vulcanizates show enhanced dispersion of filler by increased mixing time.	[96]
NBR	MWCNT, CB, conductive CB	Two roll mill	—	[97]
HNBR	MWCNT	Two roll mill method	SEM: Fine dispersion of MWCNT (20 phr) in NBR.	[99]
NBR and HNBR	MWCNT	Melt compounding	TEM/SEM: Exfoliation and intercalation of nanotubes in (H)NBR matrix.	[100]
NBR	Expanded graphite	Mechanical blending (microcomposites) and latex compounding technique	SEM: graphite disperses more uniformly with smaller lamellar agglomerates in composites.	[101]
NBR	Expanded graphite	Melt mixing	TEM: Nanoscale dispersion of graphite sheets within NBR matrix.	[102]
Carboxylic NBR	MWCNT	Solution mixing method	HRTEM: Wrinkled and folded sheets of GNS–HDA in 1 phr and 1.5 phr GNS–HDA filled XNBR composites	[104]
NBR	Graphene	Melt mixing	TEM: Composite with 10 vol % graphenes (xg-M-5) in NBR with 20 vol % softener (DOP) showed exfoliation/breakdown of the graphene nanoplatelets.	[106]
NBR	Silica/MWCNT	Melt mixing	SEM: Good dispersion of silica and CNT fillers in NBR.	[107]
TPPU-XNBR	MWCNT	Laboratory roll mill	TEM: MWCNT (3 wt %) are preferentially distributed in XNBR phase. However, 5 wt % of MWCNTs are present in TPPU phase.	[110]
TPU-NBR	MWCNT/ZnAl-LDH, CNF/ZnAl-LDH	Solution intercalation	HRTEM: 0.5 wt % SFCNT-LDH and SFCNF-LDH fillers in the blend matrix consist of uniformly distributed interconnected network.	[112]
TPU-NBR	CNF/MgAl-LDH	Solution intercalation	HRTEM: Interconnected hybrid network spread throughout in TPU/NBR/SFCNF-LDH (0.50 wt %).	[113]
TPU-NBR	MWCNT-LDH	Solution intercalation	TEM: Fine dispersion of 0.50 wt % SFCNF-LDH hybrid filler is observed in TPU/NBR matrix.	[114]
XNBR (75 phr)/SBR(25 phr)	Graphene oxide (GO)	Coagulation/roll mill	TEM: Uniform dispersion of XNBR/SBR/GO blends (GO-0.1 phr, GO-0.3 phr).	[193]
SBR/XNBR	GO	Aqueous-phase mixing (GO/SBR) and a small loading of XNBR latex, followed by co-coagulation	AFM: Full exfoliation of GO nanosheets in GO-15 sample.	[194]
NBR	Reduced graphene oxide (rGO)	Solution mixing method	TEM: rGO0.1–1 phr) appears to be evenly dispersed in the NBR matrix.	[195]

**Table 4:** Types of carbonaceous nanofillers, preparative methods adopted and morphology of silicone rubber nanocomposites.

Rubber	Filler (S)	Preparative Method	Morphology	Ref.
SR	CB, CNT	Solution mixing method	AFM: SR vulcanizates containing 3 phr of CNTs and CB show the heterogeneity of the filler and rubber components.	[116]
RTV	MWCNT	Solvent method	SEM: MWCNTs (not higher than 5 phr) is well dispersed in the silicone matrix.	[117]
HTVSR	Chitosan salt pretreated MWCNTs	Mixing method	SEM: Uniform distribution of MWCNT (4–11 wt %) in HTVSR.	[118]
SR	CB (2.5 phr)/CNT (1.0 phr)	Ball mixing	TEM: good dispersion	[119]
SR	RGO with different reduction degree	Ball milling	SEM: Graphene sheets were well dispersed in the SR matrix.	[120]
SR	MWCNT (0.3 g/50 mL solvent)	Solvent method	SEM: Rough surface texture on nanocomposite with large surface area and nanosized textures.	[121]
SR	MWCNT buckypaper	Two-step process	FESEM (fracture film): PDMS matrix fully impregnated into buckypaper network.	[122]
PDMS	CNT	Mixing method	SEM (Fracture surface) Good bonding between MWCNT (1, 2, and 4 wt %) and PDMS apparent.	[123]
SR	F-Graphene	Solvent mixing method	SEM: Uniform dispersion of CNTs (0.3 wt %) in polymer matrix.	[124]
HTVSR	CNTs pretreated by chitosan salt	Mechanical/solvent mixing	CNT (4 to 8 wt %) uniformly distributed.	[118]
PDMS	MWCNT	Twin screw extruder mixer	SEM: Good dispersibility of the tubes in the silicone matrix.	[127]
PDMS	MWCNT	By ultrasonication of mixture PDMS and silicone-g-MWCNTs in toluene.	HRTEM: Single strands of MWCNTs (0.1 wt %) disperse in silicone grafted MWCNTs.	[128]
SR	MWCNT	Two roll mixing mill	SEM: Excellent distribution of MWCNT (2, 4 and 6 phr in silicone elastomer:	[129]
SR	MWCNT	Mixing method	SEM: Good dispersion of MWCNT (1 and 3.5 wt %) reinforced SR.	[130]
RTV-SR	Graphitic nanofiller (GR):10 phr	Mixing method	AFM: completely delaminated single graphitic sheets in rubber matrix.	[131]
RTV-SR	Functionalized graphene oxide (FGO)	Solution casting	—	[132]
SR	F-Graphene	Mixing method	—	[133]
RTV-SR	Graphene (1.0 wt %)	Solvent method	SEM (Fracture surface): Graphene nanosheets randomly disperse/protrude from the fracture surface in the PDMS.	[134]
SR	Graphene nanoribbon	Solution mixing	SEM: GNR (0.4, 1.0, 2.0) distributed randomly without obvious aggregations in SR matrix	[135]
SR	Graphene nanoplatelets	liquid mixing method	FESEM: agglomeration of GNPs in the composite	[136]
SR	Triton-GNP, APTES-GNP and VTMS GNPs	Solution blending	ESM (Tensile fractured surface): VTMS-GNP and Triton-GNP seem to be well embedded in silicone matrix.	[137]
PDMS	CNF	In-situ and ex-situ	TEM: Ex-situ prepared nanocomposites feature prominent agglomeration of nanofibers in the form of lumps.	[138]
SR	MWCNT	Solvent casting	TEM/SEM: Graphene was well dispersed in the silicone rubber matrix.	[139]
VMQ	MWCNT/Graphene	Solution method	TEM: VMQ nanocomposite filled with 0.375 and 0.75 wt % MWCNT–G show better dispersion and homogeneity in VMQ matrix	[140]
SR	MWCNT-MMT	Solution blending	TEM: MWCNT/MMT (2 wt %) is well-dispersed and fully exfoliated in SR.	[141]
SR	MWCNT-Mg-Al-LDH	Solution blending	TEM: MWCNT (1 wt %) homogeneously distributed in SR.	[142]
PDMS	MWCNT/Al_2_O_3_	Ultrasonic sonication followed by casting	MWCNT-Al_2_O_3_ was well dispersed in PDMS	[143]
Liquid SR	Graphene oxide	Solution method	TEM/SEM: Random and uniform distribution of GO sheets.	[198]
Liquid silicone rubber (LSR)	Functionalized graphene oxide	Ultrasonic sonication followed by casting	SEM: TEVS-GO exhibited excellent compatibility with the LSR matrix, and formation of strong interfacial interactions between TEVS-GO and the LSR polymeric chain.	[199]
PDMS	Graphite oxide (GO) modified using 3-aminopropyltriethoxysilane (APTES)	Solvent method	—	[200]

**Table 5:** Mechanical properties of NR gums and carbon nanotubes (CNT) composites of melt and latex–based samples (Modified). Reproduced with permission from Wiley [26].

Sample	Tensile Strength (MPa)	Elongation at Break (%)	100% Modulus (MPa)
NR	14.9 ± 0.6	716 ± 52.1	0.9 ± 0.0
NR-R-CNT-5 (CNT content: 5 phr)	12.6 ± 1.4	454 ± 21.4	2.1 ± 0.0
NR-R-CNT-5 + S (Silane content was adjusted to 0.1 mL)	16.9 ± 0.7	554 ± 20.2	2.3 ± 0.1
NR-F-CNT-5 + S (Functionalized CNT: 5 phr, Silane content: 0.1 mL)	13.0 ± 0.3	476 ± 4.8	2.1 ± 0.1
N (NR latex)	16.3 ± 1.4	746 ± 42.0	0.9 ± 0.0
L-R-CNT-5 (Raw CNT content: 5 phr)	13.9 ± 0.0	525 ± 16.6	1.9 ± 0.0
L-R-CNT-5 + S (Raw CNT content: 5 phr; Silane content: 0.1 mL)	18.3 ± 0.7	595 ± 17.7	2.3 ± 0.1

**Table 6:** Mechanical properties of CNTB (carbon nanotube bundles) reinforced natural rubber composites (Modified). Reproduced with permission from Wiley [35].

Samples *	Stress at 100% (MPa)	Stress at 300% (MPa)	Tensile Strength (MPa)	Elongation at Break (%)	Tear Strength (kN/m)	Shore A Hardness
CNTB-0	2.2	8.7	24.9	659	91.8	71
CNTB-1	2.5	9.9	25.9	640	99.8	73
CNTB-3	2.7	11.2	26.5	620	103.0	74
CNTB-5	3.6	13.7	28.2	596	110.8	75

* Sample CNTB-0, CNTB-1, CNTB-3 and CNTB-5 refer to 0, 1, 3, and 5 phr of CNTB filled NR respectively.

**Table 7:** Mechanical Properties and Volume Resistivity of SBR/CB-RG(CB) Composites (Modified). Reproduced with permission from [87], Copyright Wiley, 2015.

Rubber	CB-RG(CB) Contents (CB/GO)	Tensile Strength (MPa)	Elongation at Break (%)	M200 (MPa)
SBR (100 phr)	10 phr/0 phr	3.5 ± 0.21	260 ± 10	2.3 ± 0.1
	13 phr/0 phr	3.8 ± 0.4	270 ± 20	2.4 ± 0.2
	10 phr/1 phr	4.9 ± 0.3	300 ± 20	2.8 ± 0.2
	10 phr/2 phr	4.8 ± 0.3	280 ± 20	3.1 ± 0.2
	10 phr/2 phr	4.8 ± 0.3	280 ± 20	3.1 ± 0.2

**Table 8:** Mechanical properties of acrylonitrile butadiene rubber (NBR) vulcanizates. Reproduced with permission from [97], Copyright Hindawi Publishing Corporation, 2016.

Properties	Loading (phr)	MWCNT	CCB	CB	PS
Tensile strength (MPa)	0	2.9 ± 0.37	2.9 ± 0.4	2.9 ± 0.4	2.9 ± 0.4
	5	8.6 ± 0.1	4.3 ± 0.1	4.1 ± 0.2	3.6 ± 0.4
	10	12.9 ± 0.4	9.1 ± 0.2	5.3 ± 0.5	3.8 ± 0.2
	15	17.7 ± 0.5	14.6 ± 0.6	8.5 ± 1.5	5.3 ± 0.3
Elongation at break (%)	0	182 ± 14	182 ± 14	182 ± 14	182 ± 14
	5	176 ± 2	196 ± 3	202 ± 10	210 ± 17
	10	178 ± 3	244 ± 2	217 ± 14	220 ± 1
	15	171 ± 6	277 ± 6	231 ± 14	218 ± 10
Modulus at 100% (MPa)	0	1.73 ± 0.04	1.73 ± 0.04	1.73 ± 0.04	1.73 ± 0.04
	5	5.04 ± 0.09	1.93 ± 0.02	1.82 ± 0.10	1.79 ± 0.06
	10	7.49 ± 0.56	2.47 ± 0.07	1.79 ± 0.05	1.75 ± 0.06
	15	11.28 ± 0.35	3.30 ± 0.27	2.15 ± 0.10	2.30 ± 0.12
Hardness (Shore A)	0	53 ± 0.3	53. ± 0.3	53. ± 0.3	53. ± 0.3
	5	61.8 ± 0.3	56.0 ± 0.0	55.3 ± 0.3	54.8 ± 0.8
	10	69.0 ± 0.5	61.7 ± 0.6	56.5 ± 0.9	56.8 ± 1.0
	15	69.3 ± 0.8	65.5 ± 0.5	58.2 ± 0.3	59.9 ± 0.8
Heat build-up (°C)	0	7.5 ± 0.7	7.5 ± 0.7	7.5 ± 0.7	7.5 ± 0.7
	5	11.5 ± 0.7	9.0 ± 0.0	8.0 ± 0.0	8.0 ± 0.0
	10	18.5 ± 0.7	14.0 ± 0.0	9.0 ± 0.0	9.0 ± 0.0
	15	29.0 ± 1.4	21.0 ± 0.0	10.5 ± 0.7	10.0 ± 0.0
Abrasion loss (mm^3^)	250.7 ± 10	250.7 ± 10.8	250.7 ± 10.8	250.7 ± 10.8	250.7 ± 10.8
	5	82.0 ± 12.7	91.4 ± 10.0	188.2 ± 4.7	171.2 ± 25.0
	10	54.2 ± 3.7	54.9 ± 3.1	132.6 ± 12.6	155.7 ± 8.8
	15	48.0 ± 2.6	53.3 ± 1.3	90.8 ± 6.3	127.7 ± 9.9

**Table 9:** Tensile properties of the HTVSR and the HTVSR/CNT nanocomposites. Reproduced with permission from [118], Copyright Elsevier, 2014.

Sample Name	Tensile Stress (MPa)	Tensile Strain (%)	Modulus (MPa)
Pure HTVSR hybrid	0.28	86.7	0.30
HTVSR/CNTs 4.0 wt %	0.61	144.8	0.42
HTVSR/CNTs 6.0 wt %	0.82	243.2	0.55
HTVSR/CNTs 8.0 wt %	1.59	440.1	0.55
HTVSR/CNTs 11.0 wt %	1.67	241.0	0.95

**Table 10:** Summary of mechanical properties of silicone rubber (VMQ) and its nanocomposites. Reproduced with permission from Wiley [140].

Sample	TS (MPa)	EB (%)	50% Modulus (MPa)	100% Modulus (MPa)
Neat VMQ	0.32 ± 0.02	192 ± 8	0.15	0.22
MWCNT (0.375 wt %)/VMQ	0.42 ± 0.03	113± 7	0.23	0.39
MWCNT (0.75 wt %)/VMQ	0.44 ± 0.03	104 ± 6	0.26	0.40
G (0.375 wt %)/VMQ	0.33 ± 0.01	88 ± 7	0.25	—
G (0.75 wt %)/VMQ	0.37 ± 0.01	90 ± 5	0.27	—
MWCNT-G (0.375 wt %)/VMQ	0.48 ± 0.02	144 ± 2	0.26	0.40
MWCNT-G (0.5 wt %)/VMQ	0.53 ± 0.01	158 ± 3	0.28	0.43
MWCNT-G (0.75 wt %)/VMQ	0.67 ± 0.03	194 ± 4	0.28	0.43
MWCNT-G (1.0 wt %)/VMQ	0.61 ± 0.02	165 ± 5	0.29	0.44
MWCNT-G (1.5 wt %)/VMQ	0.50 ± 0.01	123 ± 3	0.30	0.45
